# Sleep Traits and Cognitive Function: A Prospective Cohort Study With Exploration of Inflammatory Biomarkers

**DOI:** 10.1002/brb3.71514

**Published:** 2026-05-29

**Authors:** Chi Peng, Fan Yang, Fanfan Li, Ronghui Zhu, Lei Li, Zhentao Zhang, Jianyao You, Rui Wang, Yingyi Qin, Shuogui Xu

**Affiliations:** ^1^ Department of Emergency Changhai Hospital Naval Medical University Shanghai China; ^2^ Institute of Pathology and Southwest Cancer Center, Southwest Hospital Army Medical University Chongqing China; ^3^ Department of Neurology Second Affiliated Hospital of Xuzhou Medical University Xuzhou China; ^4^ Department of Health Statistics Naval Medical University Shanghai China

**Keywords:** cognitive impairment, mediation analysis, Mendelian randomization analysis, sleep traits, work shift

## Abstract

**Introduction:**

Although sleep disturbance is closely linked to cognitive impairment, the specific sleep‐related characteristics driving this deficit remain undetermined, with the mechanistic contribution of biomarkers also poorly characterized. Accumulating evidence suggests that inflammation may contribute to cognitive decline, yet its mediating role in the sleep‐cognition relationship has not been fully clarified.

**Methods:**

Based on the UK Biobank, four linear regression models (for reasoning, reaction time, visual memory, and numeric memory) and one logistic regression model (for prospective memory) were employed to analyze the relation between sleep features and cognitive impairment. Apart from that, the bootstrap mediation model and Mendelian randomization were utilized to investigate the causal association.

**Results:**

Short sleep duration (*β* = −0.394, *p* = 0.020) and long sleep duration (*β* = −0.359, *p* = 0.014) correlated with poorer numeric memory performance, while moderate sleep quality (*β* = −0.071, *p* = 0.042) and work shift were linked to impaired reasoning, reaction time, visual memory, and prospective memory. Subgroup analyses stratified by age, sex, and BMI further supported these associations. In addition, C‐reactive protein (CRP) partially mediated the snoring‐reasoning ability association (PM: *β* = −0.071, *p* = 0.049), with MR analysis confirming a causal pathway, whereas snoring elevated CRP levels to impair reasoning, accounting for 32.1% of the observed relationship.

**Conclusion:**

In this cohort study, abnormal sleep traits correlated with domain‐specific cognitive impairments, with CRP partially mediating the snoring‐reasoning association.

## 1 Introduction

Sleep plays a critical role in brain development, and is essential for human cognitive function (Girardeau and Lopes‐Dos‐Santos 2021; Mason et al. 2021). Sleep disorders have emerged as a global health concern, with primary symptoms including insomnia, circadian rhythm sleep‐wake disorders, and parasomnias (Sateia 2014; Pangaribuan et al. 2025). These disorders exert long‐term effects on health, work efficiency, and overall quality of life (Salari et al. 2023).

The relationship between sleep disorders and cognitive function remains incompletely understood. Some studies revealed that insufficient (≤ 4 h per night) or excessive (≥ 10 h per night) sleep duration was associated with impaired cognition (Y. Ma et al. 2020; Yang et al. 2024). Other research suggested that short sleep duration and higher sleep variability were significantly related to an increased incidence of cognitive impairment (Keil et al. 2023). On the contrary, several studies found a *J*‐shaped association between sleep duration and cognitive frailty, where patients with longer sleep duration exhibited a higher likelihood of cognitive frailty (Cai et al. 2025). A small sample study indicated that later sleep timing correlated with impaired global cognitive function (Gao et al. 2019; Umemura et al. 2018). Of note, the existing literature has predominantly focused on the single dimension of sleep nature, named as sleep duration. Failure to account for the comorbid nature of sleep may lead to overestimation of effect sizes. Indeed, sleep health is an inherently multidimensional event, encompassing chronotype, sleep efficiency, sleep quality, and sleep duration (Gale et al. 2024). Worse still, whether this association varies across diverse populations remained inconclusive. Existing literature indicated that age and sex substantially influenced hypothalamic structure. Furthermore, robust associations existed between these structures and region‐specific cognitive functions, with substantial cognitive decline observed in elder populations (aged 70–75 years) (Xu et al. 2025; Zhang et al. 2025).

Apart from that, most of these studies were cross‐sectional with limited sample sizes and brief follow‐up durations. In this regard, the reported associations between sleep disorders and cognition may be affected by unidentified causality. As for in‐depth mechanisms, previous studies demonstrated that inflammatory cytokines represented a potential link between sleep disorders and various neurodegenerative diseases (Irwin and Vitiello 2019; Fang et al. 2019; Irwin and Opp 2017). Sleep disturbances and short sleep duration were associated with elevated levels of systemic inflammatory markers (e.g., C‐reactive protein [CRP]), while leveraging sleep‐immune regulatory mechanisms c ould significantly reduce inflammatory cytokine levels (Irwin et al. 2016, Irwin 2019). To explore the reasons, inflammatory responses were regulated by the circadian clock and glucocorticoid levels, both of which may become dysregulated in the context of sleep disorders (Gibbs et al. 2012). Shift work, sleep deprivation, and evening chronotype were independently associated with elevated levels of circulating inflammatory markers. However, whether inflammatory cytokines mediated the association between circadian‐disrupting behaviors and cognitive function has not been evaluated (Leng et al. 2014).

In this regard, the objectives of this study were: (1) to investigate the associations between sleep disorders and cognitive function in a large, longitudinal cohort with long‐term follow‐up across overall samples and subgroups; and (2) to utilize MR to examine whether inflammatory cytokines mediated the relationship between sleep disorders and cognitive function.

## 2 Methods

### 2.1 Study Population

We conducted a prospective study utilizing the UK Biobank (UKB) database. The UKB is a large‐scale biomedical database and research resource established to investigate the prevention, diagnosis, and treatment of a wide range of diseases (Sudlow et al. 2015). The study enrolled approximately 502,128 participants between 2006 and 2010. Each participant attended one of 22 assessment centers across the UK and then completed baseline questionnaires.

In this cohort study, we excluded participants who reported baseline use of antidepressants, antipsychotic, anxiolytic, or sleep medications (Table S2). Participants with a history of neurological disorders, sleep‐related breathing disorders, or depressive disorders at baseline were also excluded. Furthermore, participants who selected “do not know” or “prefer not to answer” for items related to sleep and cognitive function metrics were excluded from the analysis.

### 2.2 Outcomes: Cognitive Function

A set of five cognitive performance tasks assessing reasoning, reaction time, visual memory, numeric memory, and prospective memory was administered via a computer touchscreen interface at baseline (T_0_, 2006–2010) and at a subsequent time point (T_1_, 2012–2013) (Supporting Information Note 1).

### 2.3 Exposure: Sleep Traits

Sleep factors included sleep duration, sleep quality, and shift work (current or former), with specific definitions for sleep and cognitive variables provided in the Tables S3 and S4.

### 2.4 Measurement of Inflammatory Biomarkers

Inflammatory factors included neutrophil count (Neutct), neutrophil percentage (NeutPct), monocyte count (MonoCt), monocyte percentage (MonoPct), lymphocyte count (LymphCt), lymphocyte percentage (LymphPct), basophil percentage (BasoPct), eosinophil percentage (EosinoPct), leukocyte count (LeukCt), CRP, neutrophil‐to‐lymphocyte ratio (NLR), Systemic immune inflammation index (SII) at baseline (M. Wang et al. 2025). The NLR was calculated as the absolute Neutct divided by the absolute LymphCt. The SII was determined using the formula: platelet count × neutrophil count/lymphocyte count. Because inflammatory markers often exhibit skewed distributions, all analyses thus used log‐transformed values for inflammatory marker levels (Supporting Information Note 2).

### 2.5 Covariates

Demographic characteristics encompassed age, sex, body mass index (BMI), socioeconomic status, race, educational level, alcohol consumption history, smoking history, and depressive symptoms (Table S1 and Supporting Information Note 3).

### 2.6 Ethics

The North West Multi‐Centre Research Ethics Committee approved the study, and all participants provided written informed consent.

### 2.7 Statistical Analyses

The five cognitive metrics assessed at time point T_1_ (2012–2013) served as outcome variables. Four linear regression models (for reasoning, reaction time, visual memory, and numeric memory) and one logistic regression model (for prospective memory) were employed. All models were adjusted for confounding factors, including cognitive indicators at baseline (T_0_) and demographic covariates (age, sex, BMI, socioeconomic status, race, educational level, alcohol consumption history, smoking history, and depressive symptoms). Results from linear regression models were reported as *β* coefficients with 95% CIs, whereas results from the logistic regression model were reported as odds ratios (ORs) with 95% CIs. To mitigate multicollinearity and identify optimal models, bidirectional stepwise regression based on the Akaike Information Criterion (AIC) was performed for each model.

Mediation analysis assessed whether inflammatory markers mediated the association between exposure variables (sleep factors) and outcomes (cognitive function). The analysis employed bootstrap resampling (1000 iterations), reporting the pure natural indirect effect (PNIE), total natural direct effect (TNDE), total effect (TE), and proportion mediated (PM) (Supporting Information Notes 4 and 5).

To investigate potential causal effects of sleep characteristics on cognitive function and the mediating role of inflammatory cytokines, a two‐sample Mendelian randomization (MR) study was conducted. Single‐nucleotide polymorphisms (SNPs), randomly allocated at conception and thus unaffected by environmental confounding, served as instrumental variables in the MR analyses. All summary‐level genome‐wide association study (GWAS) data used were publicly available. Details of the MR analysis and the specific GWAS datasets utilized are provided in Table S14 and Supporting Information Note 6.

Continuous variables were summarized as mean (SD) if normally distributed or median (interquartile range [IQR]) if nonnormally distributed. Categorical variables were presented as a number (percentage). Proportions were compared using the *χ* ^2^ test or Fisher's exact test, as appropriate. Group comparisons for continuous variables used the *t*‐test or the Wilcoxon rank‐sum test based on distributional characteristics. To enhance the robustness of our research findings, multiple imputation was used to handle missing data.

## 3 Results

### 3.1 Participant Characteristics

**FIGURE 1:**
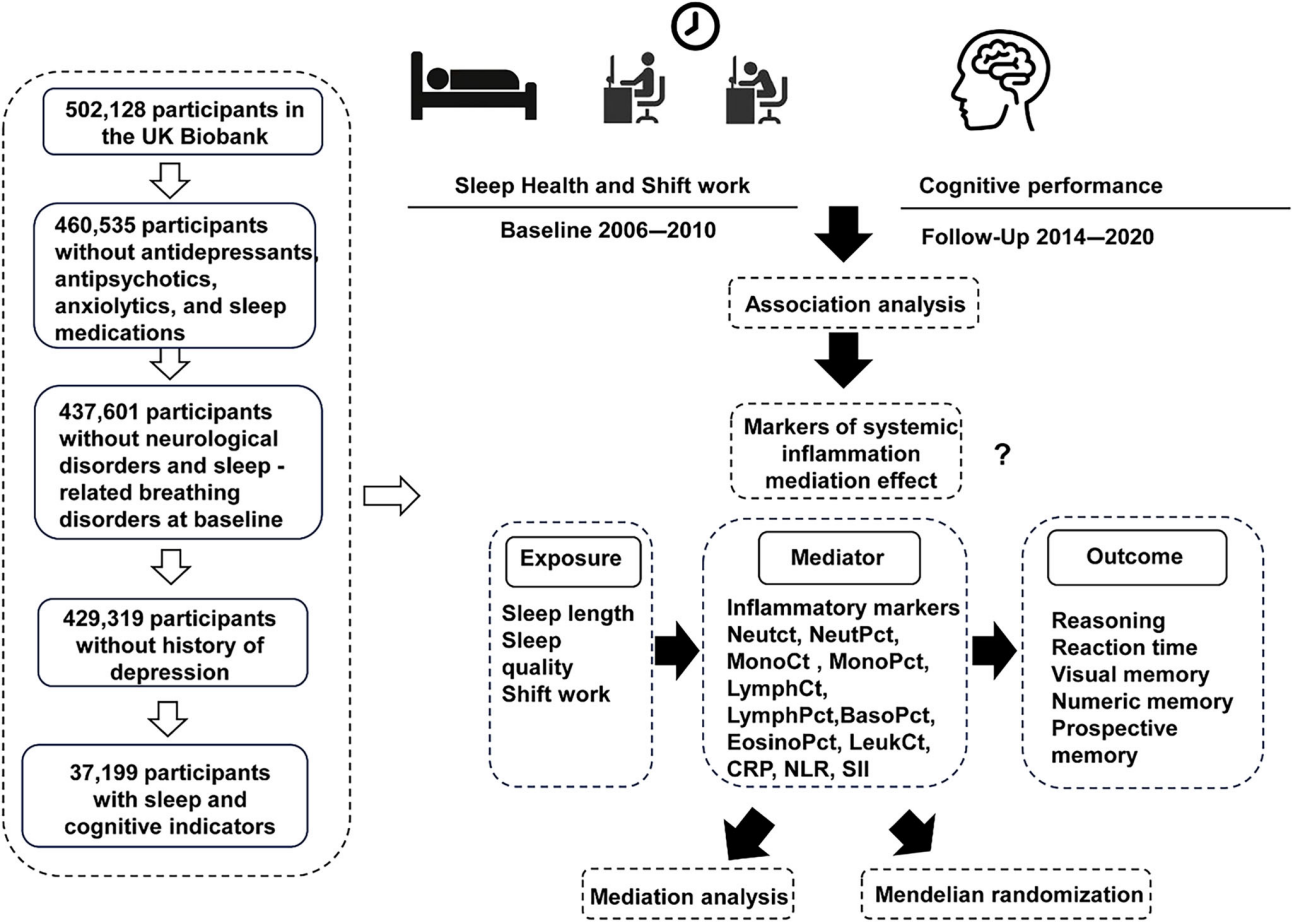
Study flowchart.

A total of 37,199 participants were included in this study (Figure 1), 51.7% of whom were female. The median age was 52.0 (47.0, 57.0). Among them, 11,774 (31.7%) participants engaged in the reasoning task, 36,995 (99.5%) participated in the reaction time task, 37,199 (100.0%) took part in the visual memory task, 2317 (6.2%) joined the numeric memory task, and 11,353 (30.5%) were involved in the prospective memory task. The baseline characteristics, sleep features, and cognitive performance at baseline and follow‐up are presented in Table 1.

**TABLE 1:** Baseline characteristics, sleep health dimensions, and shift work status along with cognitive performance.

Number of participants	37,199
Age in years (mean, SD)	52.0 (47.0, 57.0)
Sex (female, *n*, %)	19,216 (51.7)
Socioeconomic status* (mean, SD)	1.49 (1.14, 1.89)
Body mass index	25.96 (23.58, 28.82)
Race and ethnicity	
White	35,814 (96.6)
Black	287 (0.77)
Asian	572 (1.54)
Other	400 (1.08)
Educational level (college/university degree, *n*, %)	17,992 (50.7)
Depressive symptoms (*n*, %)	5234 (17.1)
Smoke, never (*n*, %)	14,181 (38.2)
Alcohol, never or previous (*n*, %)	35,621 (95.8)
Sleep length	
Short (< 6 h)	1236 (3.9)
Normal (6–8 h)	34,573 (92.9)
Long (> 8 h)	1390 (3.7)
Sleep quality score	
4–5	13,979 (37.6)
2–3	20,695 (55.6)
0–1	2525 (6.79)
Shift work	
No shift work	32,039 (86.1)
Day shift	2522 (6.78)
Mixed shift	1546 (4.16)
Night shift	461 (1.24)
Permanent night shift	631 (1.70)
Percent of cognitive function	
Reasoning	11,774 (31.7)
Reaction time	36,995 (99.5)
Visual memory	37,199 (100.0)
Numeric memory	2317 (6.2)
Prospective memory	11,353 (30.5)
Performance at baseline (mean, SD)	
Reasoning	7.00 (5.00, 8.00)
Reaction time	508 (461, 570)
Visual memory	3.00 (2.00, 5.00)
Numeric memory	7.00 (6.00, 8.00)
Prospective memory	10220 (90.0)
Performance at follow‐up (mean, SD)	
Reasoning	7.00 (5.00, 8.00)
Reaction time	573 (518, 644)
Visual memory	3.00 (2.00, 5.00)
Numeric memory	7.00 (6.00, 8.00)
Prospective memory	10,292 (90.7)

The * in Table 1 indicates statistical significance at the level of *p* <0.05.

### 3.2 The Association Between Sleep Traits and Cognitive Performance

**FIGURE 2:**
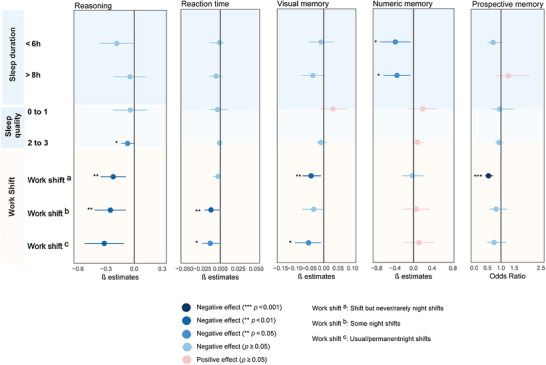
Multivariate analysis of associations between sleep traits and cognitive performance.

Complete results for all cognitive task models are presented in Tables S5–S7 and Figure 2. In the reasoning task model, after adjusting for baseline scores and other covariates, the moderate sleep quality group demonstrated inferior subsequent reasoning performance compared to the high sleep quality group (*β* = −0.071; 95% CI, −0.140 to −0.001; *p* = 0.042). Shift workers with occasional night shifts (*β* = −0.224; 95% CI, −0.359 to −0.090; *p* = 0.001), regular night shifts (*β* = −0.254; 95% CI, −0.422 to −0.090; *p* = 0.003), and prolonged night shifts (*β* = −0.320; 95% CI, −0.530 to −0.110; *p* = 0.003) showed lower reasoning scores than non‐shift workers. In subgroup analyses, the adverse effect of shift work still persisted significantly. Male participants with sleep duration < 6 h exhibited adverse effects versus those with normal sleep duration. The negative association for moderate sleep quality was observed exclusively in females, individuals > 50 years, and those with BMI 25–30 kg/m^2^. In the reaction time model, compared with non‐shift workers, shift workers with night shifts (*β* = −0.013; 95% CI, −0.022 to −0.001; *p* = 0.006) and those with prolonged night shifts (*β* = −0.014; 95% CI, −0.025 to −0.001; *p* = 0.015) showed associations with inferior subsequent task performance. In subgroup analyses, adverse associations between night shift work and task scores were observed exclusively in males, individuals > 50 years, and those with BMI 25–30 kg/m^2^. In the visual memory task model, compared with non‐shift workers, shift workers with occasional night shifts (*β* = −0.044; 95% CI, −0.074 to −0.010; *p* = 0.004) and those with prolonged night shifts (*β* = −0.053; 95% CI, −0.100 to −0.010; *p* = 0.027) emerged as predictors of inferior cognitive performance. In subgroup analyses, adverse effects of occasional night shifts were observed in females, individuals ≤ 50 years, and those with BMI 25–30 kg/m^2^. Negative associations for regular night shifts manifested in the BMI 25–30 kg/m^2^ subgroup, whereas detrimental effects of prolonged night shifts were observed in participants ≤ 50 years. Among males, moderate sleep quality independently predicted poorer performance, while sleep duration > 8 h was an adverse predictor in the BMI 25–30 kg/m^2^ group. In the digit task model, sleep duration > 8 h demonstrated an association with inferior subsequent task performance compared with moderate sleep duration (*β* = −0.319; 95% CI, −0.614 to −0.020; *p* = 0.034). Subgroup analyses showed significant adverse effects of short sleep duration among participants ≤ 50 years and long sleep duration among those > 50 years.

For the prospective memory task model, shift workers with occasional night shifts showed significantly lower task performance than non‐shift workers (OR = 0.537; 95% CI, 0.414–0.706; *p* < 0.001). The adverse effect of shift work persisted across subgroups. Significant effects of sleep duration emerged in individuals ≤ 50 years with BMI 25–30 kg/m^2^, while adverse sleep quality associations were observed in those ≤ 50 years with BMI ≤ 25 kg/m^2^ (Tables S8–S12).

### 3.3 Causal Inferences of the Effect of Sleep on Cognition

**FIGURE 3:**
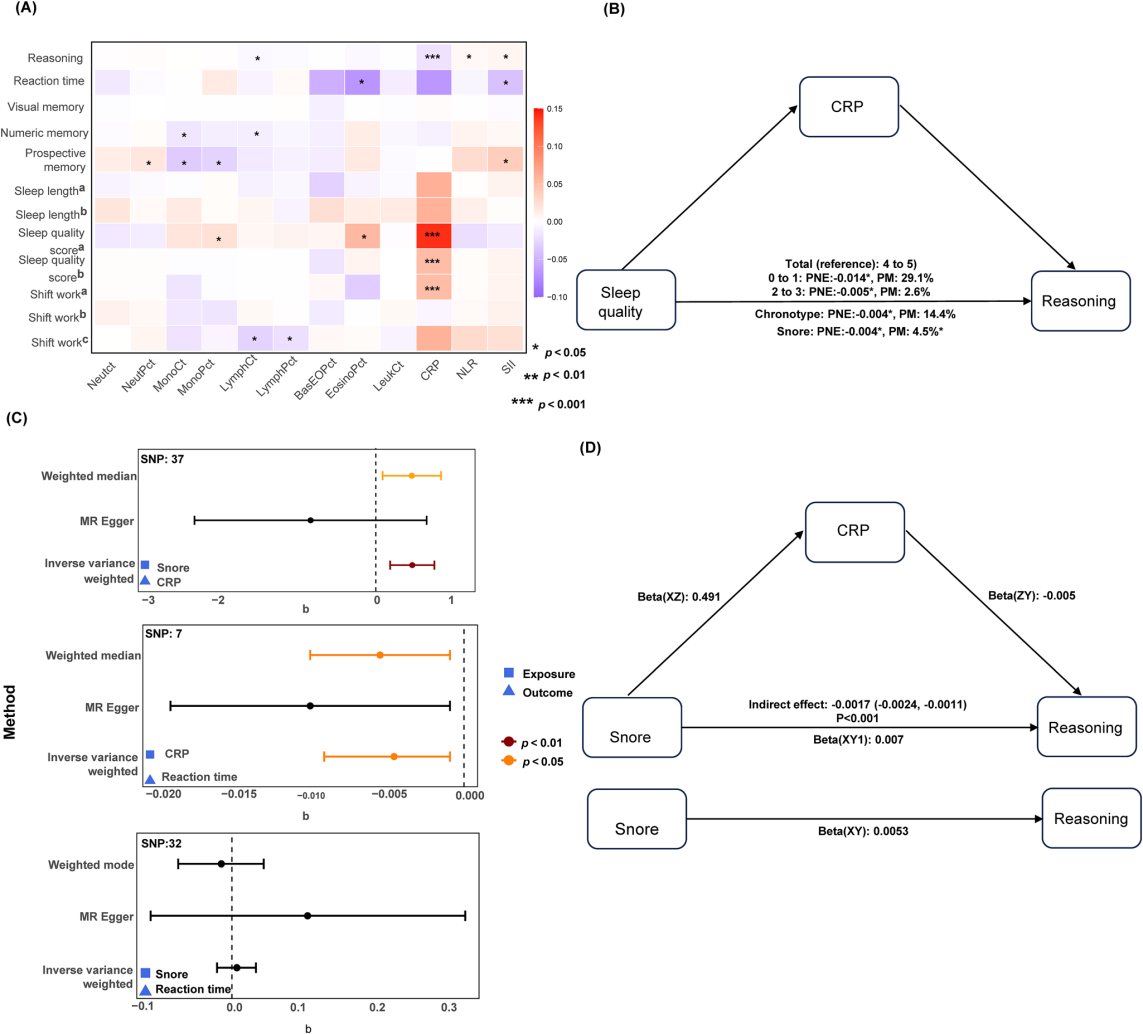
Mediating effect of inflammatory markers on sleep traits and cognitive function. (A) Summary of the correlation between inflammatory factors and outcomes of cognitive function and sleep trait phenotypes in multivariable analysis. (B) Mediational models. (C) Forest plots showed the correlation between snore, C‐reactive protein, and reaction time. (D) Mediation analysis in Mendelian randomization.

Our investigation revealed significant associations between specific inflammatory markers, sleep, and cognition. We found that LymphCt, CRP, NLR, and SII were correlated with reasoning performance. EosinoPct and SII were associated with reaction time. MonoCt and LymphCt were correlated with digit tasks. And NeutPct, MonoCt, MonoPct, and SII were correlated with prospective memory. Sleep quality had associations with MonoPct, EosinoPct, and CRP, while shift work was linked to LymphCt, LymphPct, and CRP (Table S13 and Figure 3A).

Mediation analyses established CRP as a partial mediator in the sleep quality‐reasoning association. Compared with good sleep quality, the PNIE of CRP was −0.014 (95% CI, −0.028 to −0.03) in the poor sleep quality group, accounting for 29.1% of sleep's effect on reasoning (*p* = 0.762). For moderate sleep quality, the PNIE was −0.005 (95% CI, −0.010 to −0.001), explaining 2.6% of the association (*p* = 0.360) (Table 2).

**TABLE 2:** Inflammatory markers mediating the association between sleep health dimensions, shift work status, and cognitive performance.

		PNIE		TNDE		TE		PM	
		Estimate (95% CI)	*p*‐value	Estimate (95% CI)	*p*‐value	Estimate (95% CI)	*p*‐value	Estimate (95% CI)	*p*‐value
Sleep quality and reasoning									
CRP	0–1	−0.014 (−0.028, −0.003)	0.002	−0.038 (−0.223, 0.146)	0.644	−0.052 (−0.237, 0.130)	0.556	0.291 (−1.691, 2.608)	0.762
	2–3	−0.005 (−0.010, −0.001)	0.006	−0.054 (−0.128, 0.017)	0.124	−0.059 (−0.132, 0.012)	0.092	0.026 (−0.097, 0.280)	0.360
Sleep quality and reaction time									
EosinoPct	0–1	−0.000 (−0.000, 0.000)	0.974	−0.002 (−0.013, 0.009)	0.682	−0.002 (−0.013, 0.009)	0.678	0.927 (−3.561, 4.304)	0.666
	2–3	−0.000 (−0.000, 0.000)	0.898	−0.001 (−0.005, 0.003)	0.528	−0.001 (−0.005, 0.003)	0.528	−0.001 (−0.091, 0.059)	0.932
Sleep quality and prospective memory									
MonoPct	0–1	0.999 (0.993, 1.006)	0.942	0.861 (0.586, 1.315)	0.428	0.861 (0.586, 1.313)	0.426	−0.005 (−0.244, 0.453)	0.922
	2–3	1.001 (0.997, 1.004)	0.780	0.924 (0.790, 1.088)	0.328	0.925 (0.790, 1.090)	0.332	−0.012 (−0.175, 0.142)	0.802
Sleep shift and reasoning									
CRP	Shift but never/rarely night shifts	0.001 (−0.004, 0.006)	0.752	−0.228 (−0.361, −0.095)	0.002	−0.228 (−0.362, −0.096)	0.002	−0.005 (−0.065, 0.045)	0.768
	Some night shifts	−0.001 (−0.008, 0.006)	0.814	−0.276 (−0.447, −0.105)	0.004	−0.277 (‐0.446, −0.108)	0.004	0.000 (−0.042, 0.051)	0.990
	Usual/permanent night shifts	0.002 (−0.005, 0.010)	0.620	−0.343 (−0.540, −0.121)	0.002	−0.342 (−0.537, −0.116)	0.002	−0.002 (−0.049, 0.054)	0.984
LymphPct	Shift but never/rarely night shifts	−0.001 (−0.004, 0.002)	0.660	−0.260 (−0.394, −0.136)	0.000	−0.260 (−0.394, −0.137)	0.000	−0.002 (−0.033, 0.029)	0.936
	Some night shifts	−0.002 (‐0.007, 0.002)	0.284	−0.287 (−0.443, −0.113)	0.000	−0.289 (−0.446, −0.117)	0.000	0.026 (−0.033, 0.108)	0.340
	Usual/permanent night shifts	0.001 (−0.004, 0.006)	0.770	−0.294 (−0.491, −0.084)	0.008	−0.293 (−0.491, −0.084)	0.008	−0.017 (−0.173, 0.074)	0.742
Sleep shift and numeric memory									
LymphPct	Shift but never/rarely night shifts	−0.000 (−0.013, 0.012)	0.988	0.005 (−0.235, 0.240)	0.966	0.005 (−0.238, 0.238)	0.976	−0.275 (−1.387, 2.145)	0.918
	Some night shifts	0.013 (−0.002, 0.035)	0.102	0.117 (−0.242, 0.447)	0.482	0.129 (−0.232, 0.458)	0.426	−0.276 (−4.505, 3.293)	0.902
	Usual/permanent night shifts	0.003 (−0.017, 0.028)	0.788	0.135 (−0.298, 0.520)	0.462	0.138 (−0.289, 0.531)	0.460	0.040 (−0.955, 1.031)	0.878
Sleep quality and reasoning									
CRP	Chronotype	−0.004 (−0.008, −0.001)	0.016	−0.015 (−0.083, 0.056)	0.724	−0.018 (−0.086, 0.052)	0.630	0.144 (−1.206, 1.267)	0.714
	Insomnia	−0.001 (‐0.005, 0.003)	0.620	0.046 (−0.027, 0.121)	0.214	0.045 (−0.027, 0.120)	0.226	−0.010 (−0.237, 0.174)	0.712
	Snore	−0.004 (−0.008, −0.000)	<0.001	−0.093 (−0.185, −0.012)	< 0.001	−0.097 (−0.191, −0.013)	< 0.001	0.045 (0.004, 0.422)	0.049
	Sleep length	0.001 (−0.002, 0.005)	0.456	−0.052 (−0.123, 0.025)	0.186	−0.051 (−0.123, 0.024)	0.194	−0.007 (−0.148, 0.112)	0.778
	Daytime sleepiness	0.001 (−0.008, 0.010)	0.808	−0.178 (−0.439, 0.085)	0.176	−0.177 (−0.436, 0.087)	0.176	0.002 (−0.400, 0.414)	0.974

Abbreviations: CRP, C‐reactive protein; EosinoPct, eosinophil percentage; LymphPct, lymphocyte percentage; MonoPct, monocyte percentage; PM, proportion mediated; PNIE, pure natural indirect effect; TE, total effect; TNDE, total natural direct effect.

Component‐level mediation analyses (considering sleep phenotypes, insomnia, snoring, duration, and daytime drowsiness) demonstrated significant CRP mediation for sleep phenotypes (PNIE = −0.004; 95% CI, −0.008 to −0.001; 14.4% mediated; *p* = 0.714) and snoring (PNIE = −0.004; 95% CI, −0.008 to −0.000; 4.5% mediated; *p* = 0.049) (Table 2 and Figure 3B).

### 3.4 MR Analysis

Two‐sample MR revealed significant positive causality between snoring and CRP (weighted median [WM] OR = 0.485; 95% CI, 0.091–0.879; *p* = 0.016; inverse‐variance weighted [IVW] OR = 0.491; 95% CI, 0.193–0.789; *p* = 0.001) and negative causality between CRP and reasoning ([WM] *β* = −0.006; 95% CI, −0.011 to −0.001; *p* = 0.042; [IVW] *β* = −0.005; 95% CI, −0.010 to −0.001; *p* = 0.037) (Table S15 and Figure 3C). Heterogeneity was assessed via MR‐Egger and IVW methods, while horizontal pleiotropy was evaluated by MR‐Egger and MR‐PRESSO (Table S16). Scatterplots and leave‐one‐out sensitivity analyses demonstrated robustness (Figures S1–S3).

Mediation analysis via MR indicated CRP mediated the snoring–cognition relationship: TE *β* = 0.0053; mediation effect *β* = −0.0017 (95% CI, −0.0024 to −0.0011; *p* < 0.001); PM = 32.1%. These results established CRP as a significant mediator of this causal pathway (Figure 3D).

### 3.5 Sensitivity Analysis

In the imputed dataset, we also conducted a mediation analysis, and the results were consistent with those from the original analysis (see Table S17).

### 3.6 Subgroup‐Based Mediation Effect Exploration

As for subgroup analysis, in the poor sleep quality subgroup (0–1), the PNIE of CRP was −0.004 (95% CI: −0.010 to −0.002, *p* < 0.001), while the corresponding PM showed no statistical significance (*p* = 0.600). A similar result was observed in the long sleep duration subgroup (> 8 h). Collectively, CRP did not exert a significant mediating effect on the association between sleep characteristics and reasoning ability in diverse subgroups (Tables S18 and S19).

## 4 Discussion

This observational study aimed to examine associations between sleep and cognitive function in UK adults, and to determine whether inflammatory markers partially mediated these relationships. Our findings demonstrated the adverse effects of shift work, sleep duration, and sleep quality on cognition. Specifically, shift work exhibited inverse associations with reasoning, reaction time, visual memory, and prospective memory, while sleep duration correlated negatively with digital memory. In addition, sleep quality was associated negatively with reasoning. These results aligned with previous reports, which indicated that night shift work, regardless of frequency, was related to inferior cognitive performance across domains (Ell et al. 2023; Leso et al. 2021, de Souza et al. 2025). This may be related to dual effects from sleep deprivation and circadian disruption. Shift work‐induced sleep insufficiency impaired activation in the prefrontal cortex, parietal lobe, and anterior cingulate cortex (ACC). These regions are critical for attention and executive function. Concurrently, circadian misalignment disrupted endogenous hormonal secretion and glucose metabolism, ultimately compromising neural functioning (Kecklund and Axelsson 2016; N. Ma et al. 2015; Axelsson et al. 2005). Several epidemiological studies demonstrated a *U*‐shaped relationship between sleep duration and cognitive performance, wherein both short and prolonged sleep durations were associated with impaired cognitive function (Y. Ma et al. 2020; Yang et al. 2024). Our analysis further confirmed that both short and long sleep durations imposed detrimental effects on cognitive function. Prior studies have found inverse relationships between sleep duration and β‐amyloid (Aβ) deposition, especially in early predilection sites like the ACC and medial orbitofrontal cortex (MOC) (Aslanyan et al. 2023). Sleep quality represented a multidimensional concept encompassing chronotype, insomnia, sleep duration, snoring, and daytime drowsiness. Variables including chronotype (L. Wang et al. 2024), insomnia (Zhao et al. 2022), snoring (S. Lin et al. 2024), and daytime drowsiness (Namsrai et al. 2023) were testified to be related to cognition.

Sex, age, and BMI were key covariates associated with the adverse effects of sleep disorders on cognitive function (Zhou et al. 2023; Kim et al. 2022; Mattey‐Mora and Nelson 2021). Interestingly, we found that the associations between sleep traits and cognitive function varied when stratified by subgroups of sex, age, and BMI in regression models. Studies have shown that sex can mediate cognitive function by affecting hormone secretion. Estrogen regulates melatonin synthesis, and melatonin itself exerts neuroprotective effects. Reduced melatonin levels in postmenopausal women may make them more susceptible to cognitive decline due to sleep disorders (Toffol et al. 2014). The hippocampal neurotransmitter systems (e.g., serotonin and norepinephrine) in males were more sensitive to sleep deprivation, with increased production of tryptophan metabolite kynurenine potentially inhibiting cognitive function (Baratta et al. 2018). Moreover, the stability of the biological circadian rhythm declined with aging (Hofman and Swaab 2006). Of note, the prevalence of sarcopenic obesity was increasing in the aging population, and this particular form of obesity served as a strong predictor of cognitive impairment (Peng et al. 2025). The number of patients with cerebral small vessel disease manifested as cognitive impairment was increasing, especially among the elderly population (Huang et al. 2025). A C57Bl6J mice model has revealed that high‐fat diets can induce glucocorticoid signaling alterations and memory deficits (Chakraborty et al. 2025). Moreover, further mechanistic studies have revealed that astrocyte‐derived extracellular vesicles may mediate inflammatory signaling in cognitive impairment (Wu et al. 2025).

Second, we identified CRP as a mediating inflammatory biomarker in sleep–cognition relationships. Evidence indicated that sleep modulated sympathetic nervous system (SNS) and hypothalamic–pituitary–adrenal (HPA) axis activity, thereby altering systemic inflammatory marker levels (Irwin and Cole 2011). Wiener et al. (2012) demonstrated a significant association between snoring and elevated circulating CRP levels. Snoring, clinically linked to obstructive sleep apnea hypopnea syndrome (OSAHS) may provoke intermittent hypoxia, thereby stimulating CRP production (Garvey et al. 2009; Minoguchi et al. 2005; Q. C. Lin et al. 2013).

Finally, in our primary analysis of sleep phenotypes and reasoning, the natural indirect effect (NIE) of CRP was −0.004 (*p* = 0.016), though the mediator effect did not achieve statistical significance. Mendelian mediation and subgroup mediation analysis further confirmed no significant mediation by CRP. Overall, the inflammatory pathway mediated by CRP may only weakly explain the association between chronotype and reasoning ability.

Our study provided novel insights into sleep‐cognition relationships, strengthened by its large sample size, longitudinal design, multidimensional sleep health assessment (including shift work), rigorous adjustment for confounders, and stratified subgroup analyses. The identification of CRP as a potential mediator offered clinical utility given its cost‐effectiveness and routine availability. Nevertheless, several limitations warranted acknowledgment. First, sleep parameters were assessed by self‐report, introducing possible recall bias. Second, the unavailability of precise cognitive testing timestamps and contemporaneous sleep metrics may influence performance interpretation. Third, mediation was a strict association measure. Therefore, the mediating effects of inflammatory markers on the association between sleep and cognition required further validation using in vitro experiments. Whether targeting the identified inflammatory markers can reduce the risk of cognitive impairment attributable to sleep disorders still merits further investigation.

## 5 Conclusion

Short and long sleep duration were associated with worse numeric memory, whereas poor sleep quality and shift work were related to impaired reasoning, reaction time, visual memory, and prospective memory. Notably, CRP partially and significantly mediated the association between snoring and reasoning ability, explaining 32.1% of this relationship. Mendelian randomization further confirmed that. Overall, irregular sleep patterns were related to domain‐specific cognitive decline, and CRP may act as a key mediating factor in the snoring‐related reasoning impairment pathway.

## Author Contributions

**Jianyao You**: data curation, formal analysis. **Chi Peng**: conceptualization, methodology, software, validation, investigation. Fan Yang: writing – original draft. **Lei Li**: methodology, software. **Shuogui Xu**: conceptualization, methodology, software, formal analysis, project administration, visualization, validation, investigation, writing – original draft. **Zhentao Zhang**: methodology, data curation. **Fanfan Li**: conceptualization, methodology, formal analysis, writing – original draft. **Fan Yang**: conceptualization, software, formal analysis, writing – original draft. **Ronghui Zhu**: software, methodology, conceptualization. **Rui Wang**: conceptualization, investigation, funding acquisition, visualization, validation, methodology, software. **Yingyi Qin**: conceptualization, methodology, software, formal analysis, validation, visualization, writing – original draft.

## Funding

This study received funding from the following sources: the National Key Research and Development Program of China (Grant No.: 2023YFC3107200), the Study on the Chronic Disease Governance Model of Shanghai, and the Medical Science and Technology Innovation Project of Xuzhou Municipal Health Commission (Grant No.: XWKYHT20240054).

## Consent

The authors have nothing to report.

## Conflicts of Interest

The authors declare no conflicts of interest.

## Supporting information




**Supplementary materials**: brb371514‐sup‐0001‐SuppMat.docx

## Data Availability

This research accessed data from the UK Biobank under the authorized application number 314402. These data can be found in https://www.ukbiobank.ac.uk/enable‐your‐research/apply‐for‐access.
