# Supplementary material for: Sleep Traits and Cognitive Function: A Prospective Cohort Study With Exploration of Inflammatory Biomarkers
Source: Brain Behav. 2026 May 29;16(6):e71514. doi: 10.1002/brb3.71514 (PMC13240425; doi:10.1002/brb3.71514)
Supplement: Supplementary file 1 — Supplementary materials: brb371514‐sup‐0001‐SuppMat.docx [file BRB3-16-e71514-s001.docx]

**Supplementary Online Content**

Supplementary Table 1. Table of Data-Fields

Supplementary Table 2. Table of Medication Data Fields

Supplementary Table 3. Table of Sleep Quality Composition and Calculation Rules

Supplementary Table 4. Classification of Shift Work Type​

Supplementary Table 5. Description of Genetic Instruments Used in the Study​

Supplementary Table 6. Multivariate Analysis of Associations Between Sleep Length and Cognitive Performance​

Supplementary Table 7. Multivariate Analysis of Associations Between Sleep Quality and Cognitive Performance​

Supplementary Table 8. Multivariate Analysis of Associations Between Work Shift and Cognitive Performance​

Supplementary Table 9. Multivariate Analysis of Associations Between Sleep Health Dimensions, and Shift Work Status and Reasoning Stratified by Sex, Age and BMI​

Supplementary Table 10. Multivariate Analysis of Associations Between Sleep Health Dimensions, and Shift Work Status with Reaction Time Stratified by Sex, Age and BMI​

Supplementary Table 11. Multivariate Analysis of Associations Between Sleep Health Dimensions, and Shift Work Status with Visual Memory Stratified by Sex, Age and BMI​

Supplementary Table 12. Multivariate Analysis of Associations Between Sleep Health Dimensions, and Shift Work Status with Numeric Memory Stratified by Sex, Age and BMI​

Supplementary Table 13. Multivariate Analysis of Associations Between Sleep Health Dimensions, and Shift Work Status with Prospective Memory Stratified by Sex, Age and BMI​

Supplementary Table 14. Correlation Between Inflammatory Factors and Outcomes of Cognitive Function and Sleep-Related Factors in Multivariable Analysis​

Supplementary Table 15. Two-Sample MR Analysis Results​

Supplementary Table 16. Sensitivity Analysis, Including Heterogeneity Test and Horizontal Pleiotropy Test​

Supplementary Table 17. Inflammatory Markers Mediating the Association Between Sleep Health Dimensions, Shift Work Status and Cognitive Performance (Imputed Dataset)​

Supplementary table 18. Correlation Between Inflammatory Factors and Outcomes of Cognitive Function and Sleep-Related Factors in Multivariable Analysis Stratified by Sex, Age and BMI

Supplementary table 19. Inflammatory Markers Mediating the Association Between Sleep Health Dimensions, Shift Work Status and Cognitive Performance Stratified by Sex, Age and BMI

Supplementary Figure 1. Scatter Plots of Genetic Correlation Between Sleep Quality, CRP and Reasoning by Different MR Analysis Methods​

Supplementary Figure 2. "Leave-One-Out" Results of Genetic Correlation Between Sleep Quality, CRP and Reasoning by Different MR Analysis Methods​

Supplementary Figure 3. "Leave-One-Out" Results of Genetic Correlation Between Sleep Quality, CRP and Reasoning by Different MR Analysis Methods​

Supplementary Note 1. Details of UK Biobank Assessment​

Supplementary Note 2. Details of Inflammation-Related Biomarkers​

Supplementary Note 3. Details of Covariates​

Supplementary Note 4. Indicators of Mediation Analysis​

Supplementary Note 5. Details of Mediation Analysis in Mendelian Randomization​

Supplementary Note 6. Details of the Mendelian Randomization

| **Supplementary Table 1. Table of Data-Fields** | |
| --- | --- |
| Variables | Data-Field code |
| **Baseline characteristics** |  |
| Age | 21022 |
| Smoking status | 20116 |
| Ethnic | 21000 |
| Education | 6138 |
| BMI | 21001 |
| Alcohol drinker status intake | 20117 |
| Physical activity | 22040 |
| Education | 6138 |
| Townsend deprivation index | 22189 |
| **Inflammation markers** |  |
| Neutrophill count | 30140 |
| Neutrophill percentage | 30200 |
| Monocyte count | 30130 |
| Monocyte percentage | 30190 |
| Lymphocyte count | 30120 |
| Lymphocyte percentage | 30180 |
| Basophill count | 30160 |
| Eosinophill count | 30150 |
| White blood cell (leukocyte) count | 30000 |
| C-reactive protein | 30710 |
| **Sleep traits** |  |
| **Sleep length** | 1160 |
| **Sleep quality** |  |
| chronotype | 1180 |
| insomnia | 1200 |
| snore | 1210 |
| daytime dozing | 1220 |
| **Work shift** |  |
| Job involves shift work | 826 |
| Job involves night shift work | 3426 |
| **Congtive performance** |  |
| Reasoning | 20016 |
| Reaction time | 20023 |
| Visual memory | 399 |
| Numeric memory | 4282 |
| prospective memory | 20018 |

| **Supplementary Table 2. Table of** **Medication Data Fields** | | |
| --- | --- | --- |
| Variables | Data-Field code | Variable code |
| Sleep medications | 20003 | 1140863144;1140863152;1140863202;1140865016; 1140863182;1140863302;1140883656;1140928004; 1140862810;1140882082;1140879730;1140863286; 1140863442;1140863120;1140863328;1140863176; 1140863292;1140864916;1140863152;1140863454; 1140863308;1140863350;1140863310;1140863112; 1140909798;1140863378;1140863028;1140867938; 1140863110;1140863036;1140863372;1140867136; 1140882312;1140855870;1140855832;1140875434; 1140875436;1140863410;1140863016;1140855824; 1140855890;1140856040;1140863194;1140863106; 1140867668 |
| Anti-depressants | 20003 | 1140863442;1140863378;1140863372;1140863318; 1140863302;1140863268;1140868274;1140863262; 1140863202;1140863182;1140863176;1141157496; 1140863152;1140863144;1140863140;1140856040; 1140855944;1140855914;1140855860;1140855824; 1140909798;1141171404;1141180444 |
| Anti-psychotics | 20003 | 1140868170;1140928916;1141152848;1140867444; 1140879658;1140868120;1140867944;1141153490; 1140867304;1141152860;1140867168;1141195974; 1140867244;1140867152;1140909800;1140867420; 1140879746;1141177762;1140867456;1140867150; 1141167976;1140882100;1140867342;1140863416; 1141202024;1140882098;1140867184;1140867092; 1140882320;1140910358;1140867208;1140909802; 1140867134;1140867306;1140867210;1140882098;  1140867078;1140867218;1141201792;1141200458; 1140867136;1140879750;1140867180;1140867546; 1140856416;1140856418;1140856420;1140927956; |
| Anxiolytics | 20003 | 1140867938;1140867948;1140879616;1140921600; 1140879540;1140867878;1140916282;1140909806; 1140867888;1141152732;1141180212;1140879634; 1140867876;1140882236;1141190158;1141200564; 1140867726;1140879620;1140867818;1140867940; 1140867942;1140879630;1140879628;1141151946; 1140867624;1140867756;1140867884;1141151978; 1141152736;1141201834;1140867690;1141168396; 1140867640;1140867920;1140867850;1140879544; 1141200570;1140867934;1140867914;1140867944; 1141151982;1140882244;1140879556;1140867852; 1140867914;1140867944;1140868120;1140867784; 1140867812;1140867668;1140867940 |

| **Supplementary Table 3. Table of Sleep Quality Composition and Calculation Rules** | | |
| --- | --- | --- |
| Variables | Definition |  |
| Chronotype | "Definitely a morning person" or "more a morning than evening person" is scored 1 point, while other cases are scored 0 point. | According to this cumulative value, the sleep quality is classified into three categories: "healthy" (4 to 5 points),  "moderate" (2 to 3 points), and "poor" (0 to 1 point). |
| Insomnia | If participants answer "never/seldom", they will score 1 point for the factor of insomnia; if they answer "sometimes" or "usually", they will be regarded as having frequent insomnia symptoms  and will score 0 point. |  |
| Snore | If participant indicate that they have never or rarely snored, they will be assigned 1 point for the factor of snoring; if a participant sometimes or often snores, they will instead be given 0 points. |  |
| Sleep duration | If one sleeps for 7 to 8 hours a day, they will be scored 1 point, while for other durations of sleep, they will be scored 0 point. |  |
| Daytime dozing | If participants indicate that they have never or rarely fallen asleep involuntarily during the daytime, meaning they have no problem  of daytime sleepiness, they will receive 1 point. However, if participants sometimes or often dozes off or falls asleep unconsciously during the daytime, they will be judged to have  the problem of daytime sleepiness and thus receive 0 points for this factor. |  |

| **Supplementary Table 4. Classification of Shift Work Type** | | |
| --- | --- | --- |
| Question 1: Does your work involve shift work? | Question 2: Does your job involve night shifts? | Shift work type |
| Sometimes | Never/rarely | Shift but never/rarely night shifts |
|  | Sometime | Some night shifts |
|  | Usually | Usual/permanentnight shifts |
|  | Always | Usual/permanentnight shifts |
| Usually | Never/rarely | Shift but never/rarely night shifts |
|  | Sometime | Some night shifts |
|  | Usually | Usual/permanentnight shifts |
|  | Always | Usual/permanentnight shifts |
| Always | Never/rarely | Shift but never/rarely night shifts |
|  | Sometime | Some night shifts |
|  | Usually | Usual/permanent night shifts |
|  | Always | Usual/permanent night shifts |

**Supplementary table 5. Description of genetic instruments used in the study**

| Factor | Dataset | Sample size | Population |
| --- | --- | --- | --- |
| Chronotype | ukb-b-4956 | 413,343 | European |
| Insomnia | ukb-b-3957 | 462,341 | European |
| Snore | ebi-a-GCST009760 | 408,317 | European |
| Sleep duration | ukb-b-4424 | 460,099 | European |
| Daytime dozing | ukb-b-5776 | 460,913 | European |
| CRP | ieu-b-35 | 204,402 | European |
| Reasoning | ukb-b-4323 | 151,059 | European |

| **Supplementary Table 6. Multivariate Analysis of Associations Between Sleep duration and Cognitive Performance** | | | |
| --- | --- | --- | --- |
|  | **Sleep duration** | **β (95% CI)** | ***P* value** |
| Reasoning | <6h | -0.186 (-0.373, 0.000) | 0.051 |
|  | >8 | -0.045 (-0.219, 0.130) | 0.614 |
| Reaction time | <6h | -0.001 (-0.015, 0.005) | 0.357 |
|  | >8 | -0.006 (-0.015, 0.003) | 0.216 |
| Visual memory | <6h | -0.009 (-0.052, 0.035) | 0.700 |
|  | >8 | -0.038 (-0.077, 0.002) | 0.063 |
| Numeric memory | <6h | -0.394 (-0.725, -0.063) | 0.020 |
|  | >8 | -0.359 (-0.651, -0.066) | 0.016 |
|  | **Sleep duration** | **OR (95% CI)** | ***P* value** |
| Prospective memory | <6h | 0.707 (0.490, 1.050) | 0.074 |
|  | >8 | 1.282 (0.830, 2.090) | 0.289 |

CI, Confidence Interval; OR, Odds Ratio;

| **Supplementary table 7. Multivariate Analysis of Associations Between Sleep quality and Cognitive Performance** | | | |
| --- | --- | --- | --- |
|  | **Sleep quality** | **β (95% CI)** | ***P* value** |
| Reasoning | 0 to 1 | -0.042 (-0.226, 0.140) | 0.656 |
|  | 2 to 3 | -0.071 (-0.140, -0.001) | 0.042 |
| Reaction time | 0 to 1 | -0.004 (-0.015, 0.010) | 0.408 |
|  | 2 to 3 | -0.001 (-0.005, 0.000) | 0.522 |
| Visual memory | 0 to 1 | 0.032 (-0.012, 0.080) | 0.153 |
|  | 2 to 3 | -0.010 (-0.025, 0.010) | 0.205 |
| Numeric memory | 0 to 1 | 0.198 (-0.109, 0.500) | 0.206 |
|  | 2 to 3 | 0.084 (-0.038, 0.210) | 0.178 |
|  | **Sleep quality** | **OR (95% CI)** | ***P* value** |
| Prospective memory | 0 to 1 | 0.950 (0.636, 1.464) | 0.808 |
|  | 2 to 3 | 0.930 (0.788, 1.096) | 0.388 |

CI, Confidence Interval; OR, Odds Ratio;

| **Supplementary table 8. Multivariate Analysis of Associations Between Work Shift and Cognitive Performance** | | | |
| --- | --- | --- | --- |
|  | **Work Shift** | **β (95% CI)** | ***P* value** |
| Reasoning | Shift but never/rarely night shifts | -0.224 (-0.359, -0.090) | 0.001 |
|  | Some night shifts | -0.254 (-0.422, -0.090) | 0.003 |
|  | Usual/permanent night shifts | -0.320 (-0.530, -0.110) | 0.003 |
| Reaction time | Shift but never/rarely night shifts | -0.003 (-0.01, 0.000) | 0.399 |
|  | Some night shifts | -0.013 (-0.022, -0.001) | 0.006 |
|  | Usual/permanent night shifts | -0.014 (-0.025, -0.001) | 0.015 |
| Visual memory | Shift but never/rarely night shifts | -0.044 (-0.074, -0.010) | 0.004 |
|  | Some night shifts | -0.035 (-0.074, 0.000) | 0.074 |
|  | Usual/permanent night shifts | -0.053 (-0.100, -0.010) | 0.027 |
| Numeric memory | Shift but never/rarely night shifts | -0.020 (-0.248, 0.210) | 0.867 |
|  | Some night shifts | 0.062 (-0.234, 0.360) | 0.682 |
|  | Usual/permanent night shifts | 0.117 (-0.224, 0.460) | 0.502 |
|  | **Work Shift** | **OR (95% CI)** | ***P* value** |
| Prospective memory | Shift but never/rarely night shifts | 0.537 (0.414, 0.706) | <0.001 |
|  | Some night shifts | 0.822 (0.567, 1.230) | 0.320 |
|  | Usual/permanent night shifts | 0.741 (0.478, 1.203) | 0.201 |

CI, Confidence Interval; OR, Odds Ratio;

| **Supplementary table 9.** **Multivariate Analysis of Associations Between Sleep Health Dimensions, and Shift Work Status and Reasoning Stratified by Sex, Age and BMI** | | | | | | | | | |
| --- | --- | --- | --- | --- | --- | --- | --- | --- | --- |
|  | **Sleep length** | **β (95%CI)** | **P value** | **Sleep quality** | **β (95%CI)** | **P value** | **Work Shift** | **β (95%CI)** | **P value** |
| **Sex** |  |  |  |  |  |  |  |  |  |
| Males | <6h | -0.307 (-0.573, -0.041) | 0.024 | 0 to 1 | -0.073 (-0.320, 0.174) | 0.562 | Shift but never/rarely night shifts | -0.231 (-0.440, -0.021) | 0.031 |
|  | >8 | 0.137 (-0.144, 0.417) | 0.339 | 2 to 3 | -0.014 (-0.116, 0.087) | 0.781 | Some night shifts | -0.332 (-0.557, -0.107) | 0.004 |
|  |  |  |  |  |  |  | Usual/permanent night shifts | -0.274 (-0.539, -0.008) | 0.044 |
| Females | <6h | -0.060 (-0.323, 0.203) | 0.655 | 0 to 1 | -0.1 (-0.379, 0.179) | 0.482 | Shift but never/rarely night shifts | -0.239 (-0.414, -0.065) | 0.007 |
|  | >8 | -0.162 (-0.380, 0.056) | 0.145 | 2 to 3 | -0.147 (-0.239, -0.054) | 0.002 | Some night shifts | -0.185 (-0.442, 0.072) | 0.158 |
|  |  |  |  |  |  |  | Usual/permanent night shifts | -0.463 (-0.813, -0.114) | 0.009 |
| **Age** |  |  |  |  |  |  |  |  |  |
| ≤50 | <6h | -0.286 (-0.599, 0.027) | 0.074 | 0 to 1 | 0.019 (-0.294, 0.333) | 0.904 | Shift but never/rarely night shifts | -0.184 (-0.387, 0.019) | 0.075 |
|  | >8 | -0.172 (-0.428, 0.084) | 0.189 | 2 to 3 | -0.013 (-0.119, 0.093) | 0.815 | Some night shifts | -0.242 (-0.48, -0.003) | 0.047 |
|  |  |  |  |  |  |  | Usual/permanent night shifts | -0.196 (-0.474, 0.083) | 0.168 |
| >50 | <6h | -0.153 (-0.386, 0.080) | 0.198 | 0 to 1 | -0.142 (-0.371, 0.087) | 0.224 | Shift but never/rarely night shifts | -0.274 (-0.453, -0.094) | 0.003 |
|  | >8 | 0.076 (-0.158, 0.310) | 0.525 | 2 to 3 | -0.126 (-0.216, -0.036) | 0.006 | Some night shifts | -0.298 (-0.533, -0.063) | 0.013 |
|  |  |  |  |  |  |  | Usual/permanent night shifts | -0.524 (-0.838, -0.21) | 0.001 |
| **BMI CATEGORY** | |  |  |  |  |  |  |  |  |
| ≤25 | <6h | -0.280 (-0.616, 0.056) | 0.102 | 0 to 1 | -0.12 (-0.469, 0.229) | 0.501 | Shift but never/rarely night shifts | -0.402 (-0.62, -0.184) | 0.000 |
|  | >8 | -0.092 (-0.368, 0.183) | 0.510 | 2 to 3 | -0.101 (-0.208, 0.006) | 0.063 | Some night shifts | -0.313 (-0.635, 0.009) | 0.057 |
|  |  |  |  |  |  |  | Usual/permanent night shifts | -0.538 (-0.908, -0.167) | 0.004 |
| 25-<30 | <6h | -0.181 (-0.460, 0.098) | 0.204 | 0 to 1 | -0.039 (-0.325, 0.247) | 0.789 | Shift but never/rarely night shifts | -0.152 (-0.351, 0.047) | 0.134 |
|  | >8 | 0.071 (-0.205, 0.347) | 0.615 | 2 to 3 | -0.118 (-0.222, -0.013) | 0.027 | Some night shifts | -0.185 (-0.428, 0.058) | 0.135 |
|  |  |  |  |  |  |  | Usual/permanent night shifts | -0.301 (-0.604, 0.003) | 0.052 |
| ≥30 | <6h | -0.172 (-0.549, 0.205) | 0.371 | 0 to 1 | -0.092 (-0.434, 0.25) | 0.596 | Shift but never/rarely night shifts | -0.072 (-0.407, 0.262) | 0.671 |
|  | >8 | -0.155 (-0.533, 0.223) | 0.422 | 2 to 3 | 0.057 (-0.115, 0.229) | 0.514 | Some night shifts | -0.383 (-0.722, -0.044) | 0.027 |
|  |  |  |  |  |  |  | Usual/permanent night shifts | -0.148 (-0.606, 0.311) | 0.528 |

CI, Confidence Interval; BMI, Body Mass Index;

| **Supplementary table 10. Multivariate Analysis of Associations Between Sleep Health Dimensions, and Shift Work Status with Reaction Time Stratified by Sex, Age and BMI** | | | | | | | | | |
| --- | --- | --- | --- | --- | --- | --- | --- | --- | --- |
|  | **Sleep length** | **β (95%CI)** | ***P* value** | **Sleep quality** | **β (95%CI)** | ***P* value** | **Work Shift** | **β (95%CI)** | **P value** |
| **Sex** |  |  |  |  |  |  |  |  |  |
| Males | <6h | 0.001 (-0.013, 0.016) | 0.840 | 0 to 1 | -0.008 (-0.021, 0.006) | 0.262 | Shift but never/rarely night shifts | -0.001 (-0.012, 0.009) | 0.820 |
|  | >8 | -0.005 (-0.019, 0.010) | 0.545 | 2 to 3 | -0.002 (-0.007, 0.003) | 0.370 | Some night shifts | -0.021 (-0.032, -0.009) | 0.001 |
|  |  |  |  |  |  |  | Usual/permanentnight shifts | -0.014 (-0.027, 0) | 0.051 |
| Females | <6h | -0.012 (-0.026, 0.002) | 0.105 | 0 to 1 | -0.004 (-0.02, 0.013) | 0.665 | Shift but never/rarely night shifts | -0.005 (-0.015, 0.004) | 0.268 |
|  | >8 | -0.007 (-0.018, 0.005) | 0.260 | 2 to 3 | -0.001 (-0.006, 0.004) | 0.722 | Some night shifts | -0.004 (-0.018, 0.01) | 0.583 |
|  |  |  |  |  |  |  | Usual/permanentnight shifts | -0.018 (-0.036, 0) | 0.055 |
| **Age** |  |  |  |  |  |  |  |  |  |
| ≤50 | <6h | -0.008 (-0.024, 0.009) | 0.364 | 0 to 1 | -0.006 (-0.022, 0.01) | 0.457 | Shift but never/rarely night shifts | 0 (-0.01, 0.011) | 0.930 |
|  | >8 | -0.001 (-0.014, 0.012) | 0.901 | 2 to 3 | -0.001 (-0.006, 0.004) | 0.743 | Some night shifts | -0.007 (-0.019, 0.005) | 0.259 |
|  |  |  |  |  |  |  | Usual/permanentnight shifts | -0.012 (-0.026, 0.003) | 0.112 |
| >50 | <6h | -0.004 (-0.017, 0.009) | 0.561 | 0 to 1 | -0.006 (-0.02, 0.007) | 0.377 | Shift but never/rarely night shifts | -0.006 (-0.016, 0.004) | 0.216 |
|  | >8 | -0.010 (-0.023, 0.003) | 0.116 | 2 to 3 | -0.002 (-0.007, 0.002) | 0.342 | Some night shifts | -0.015 (-0.028, -0.002) | 0.026 |
|  |  |  |  |  |  |  | Usual/permanentnight shifts | -0.013 (-0.03, 0.004) | 0.130 |
| **BMI CATEGORY** | |  |  |  |  |  |  |  |  |
| ≤25 | <6h | -0.006 (-0.024, 0.012) | 0.500 | 0 to 1 | -0.002 (-0.022, 0.017) | 0.811 | Shift but never/rarely night shifts | -0.011 (-0.022, 0.001) | 0.065 |
|  | >8 | -0.010 (-0.024, 0.005) | 0.191 | 2 to 3 | -0.001 (-0.006, 0.004) | 0.733 | Some night shifts | 0.006 (-0.01, 0.021) | 0.485 |
|  |  |  |  |  |  |  | Usual/permanentnight shifts | -0.016 (-0.035, 0.004) | 0.113 |
| 25-<30 | <6h | 0.000 (-0.016, 0.016) | 0.998 | 0 to 1 | -0.012 (-0.028, 0.004) | 0.135 | Shift but never/rarely night shifts | -0.005 (-0.016, 0.006) | 0.359 |
|  | >8 | -0.002 (-0.017, 0.013) | 0.789 | 2 to 3 | -0.005 (-0.011, 0.001) | 0.076 | Some night shifts | -0.028 (-0.042, -0.015) | 0.000 |
|  |  |  |  |  |  |  | Usual/permanentnight shifts | -0.014 (-0.03, 0.002) | 0.088 |
| ≥30 | <6h | -0.013 (-0.033, 0.008) | 0.220 | 0 to 1 | 0 (-0.019, 0.02) | 0.973 | Shift but never/rarely night shifts | 0.014 (-0.003, 0.03) | 0.100 |
|  | >8 | -0.007 (-0.028, 0.014) | 0.504 | 2 to 3 | 0.005 (-0.004, 0.014) | 0.283 | Some night shifts | -0.011 (-0.03, 0.008) | 0.272 |
|  |  |  |  |  |  |  | Usual/permanentnight shifts | -0.016 (-0.039, 0.007) | 0.170 |

CI, Confidence Interval; BMI, Body Mass Index;

| **Supplementary table 11. Multivariate Analysis of Associations Between Sleep Health Dimensions, and Shift Work Status with Visual Memory Stratified by Sex, Age and BMI** | | | | | | | | | |
| --- | --- | --- | --- | --- | --- | --- | --- | --- | --- |
|  | **Sleep length** | **β (95%CI)** | ***P* value** | **Sleep quality** | **β (95%CI)** | ***P* value** | **Work Shift** | **β (95%CI)** | ***P* value** |
| **Sex** |  |  |  |  |  |  |  |  |  |
| Males | <6h | 0.001 (-0.063, 0.064) | 0.987 | 0 to 1 | 0.054 (-0.004, 0.111) | 0.069 | Shift but never/rarely night shifts | -0.042 (-0.088, 0.004) | 0.072 |
|  | >8 | -0.027 (-0.091, 0.038) | 0.422 | 2 to 3 | -0.024 (-0.046, -0.001) | 0.037 | Some night shifts | -0.041 (-0.093, 0.011) | 0.124 |
|  |  |  |  |  |  |  | Usual/permanentnight shifts | -0.048 (-0.108, 0.012) | 0.115 |
| Females | <6h | -0.017 (-0.077, 0.044) | 0.592 | 0 to 1 | -0.018 (-0.085, 0.05) | 0.609 | Shift but never/rarely night shifts | -0.047 (-0.087, -0.008) | 0.020 |
|  | >8 | -0.041 (-0.090, 0.009) | 0.106 | 2 to 3 | 0.004 (-0.017, 0.024) | 0.723 | Some night shifts | -0.034 (-0.092, 0.024) | 0.250 |
|  |  |  |  |  |  |  | Usual/permanentnight shifts | -0.067 (-0.144, 0.010) | 0.088 |
| **Age** |  |  |  |  |  |  |  |  |  |
| ≤50 | <6h | 0.013 (-0.059, 0.085) | 0.730 | 0 to 1 | 0.016 (-0.052, 0.084) | 0.645 | Shift but never/rarely night shifts | -0.071 (-0.116, -0.026) | 0.002 |
|  | >8 | -0.035 (-0.092, 0.022) | 0.228 | 2 to 3 | -0.019 (-0.041, 0.004) | 0.109 | Some night shifts | -0.024 (-0.076, 0.029) | 0.378 |
|  |  |  |  |  |  |  | Usual/permanentnight shifts | -0.077 (-0.139, -0.014) | 0.016 |
| >50 | <6h | -0.022 (-0.077, 0.033) | 0.427 | 0 to 1 | 0.032 (-0.025, 0.088) | 0.275 | Shift but never/rarely night shifts | -0.026 (-0.066, 0.015) | 0.214 |
|  | >8 | -0.037 (-0.092, 0.017) | 0.178 | 2 to 3 | -0.006 (-0.026, 0.015) | 0.576 | Some night shifts | -0.038 (-0.094, 0.018) | 0.187 |
|  |  |  |  |  |  |  | Usual/permanentnight shifts | -0.013 (-0.083, 0.058) | 0.724 |
| **BMI category** | |  |  |  |  |  |  |  |  |
| ≤25 | <6h | -0.017 (-0.096, 0.063) | 0.682 | 0 to 1 | 0.056 (-0.029, 0.141) | 0.195 | Shift but never/rarely night shifts | -0.045 (-0.094, 0.004) | 0.074 |
|  | >8 | -0.003 (-0.065, 0.060) | 0.933 | 2 to 3 | -0.001 (-0.025, 0.022) | 0.930 | Some night shifts | 0.005 (-0.064, 0.075) | 0.883 |
|  |  |  |  |  |  |  | Usual/permanentnight shifts | -0.012 (-0.097, 0.072) | 0.777 |
| 25-<30 | <6h | -0.025 (-0.091, 0.041) | 0.455 | 0 to 1 | -0.035 (-0.102, 0.032) | 0.304 | Shift but never/rarely night shifts | -0.06 (-0.106, -0.014) | 0.010 |
|  | >8 | -0.079 (-0.142, -0.016) | 0.014 | 2 to 3 | -0.009 (-0.033, 0.014) | 0.437 | Some night shifts | -0.057 (-0.114, -0.001) | 0.048 |
|  |  |  |  |  |  |  | Usual/permanentnight shifts | -0.069 (-0.139, 0) | 0.051 |
| ≥30 | <6h | 0.022 (-0.062, 0.106) | 0.608 | 0 to 1 | 0.088 (-0.008, 0.168) | 0.130 | Shift but never/rarely night shifts | -0.017 (-0.085, 0.051) | 0.617 |
|  | >8 | -0.023 (-0.110, 0.063) | 0.596 | 2 to 3 | -0.028 (-0.065, 0.01) | 0.152 | Some night shifts | -0.058 (-0.137, 0.021) | 0.147 |
|  |  |  |  |  |  |  | Usual/permanentnight shifts | -0.082 (-0.177, 0.014) | 0.093 |

CI, Confidence Interval; BMI, Body Mass Index;

| **Supplementary table 12. Multivariate Analysis of Associations Between Sleep Health dimensions, and Shift Work Status with Numeric Memory Stratified by Sex, Age and BMI** | | | | | | | | | |
| --- | --- | --- | --- | --- | --- | --- | --- | --- | --- |
|  | **Sleep length** | **β (95%CI)** | ***P* value** | **Sleep quality** | **β (95%CI)** | ***P* value** | **Work Shift** | **β (95%CI)** | ***P* value** |
| **Sex** |  |  |  |  |  |  |  |  |  |
| Males | <6h | -0.403 (-0.889, 0.083) | 0.104 | 0 to 1 | 0.314 (-0.111, 0.738) | 0.147 | Shift but never/rarely night shifts | -0.039 (-0.400, 0.322) | 0.831 |
|  | >8 | -0.479 (-0.995, 0.037) | 0.069 | 2 to 3 | 0.226 (-0.047, 0.405) | 0.130 | Some night shifts | 0.136 (-0.260, 0.532) | 0.500 |
|  |  |  |  |  |  |  | Usual/permanentnight shifts | 0.162 (-0.253, 0.577) | 0.444 |
| Females | <6h | -0.347 (-0.798, 0.105) | 0.132 | 0 to 1 | 0.034 (-0.409, 0.477) | 0.880 | Shift but never/rarely night shifts | 0.035 (-0.255, 0.325) | 0.812 |
|  | >8 | -0.316 (-0.665, 0.032) | 0.075 | 2 to 3 | -0.101 (-0.268, 0.066) | 0.234 | Some night shifts | -0.072 (-0.511, 0.367) | 0.747 |
|  |  |  |  |  |  |  | Usual/permanentnight shifts | -0.021 (-0.645, 0.603) | 0.947 |
| **Age** |  |  |  |  |  |  |  |  |  |
| ≤50 | <6h | -0.957 (-1.511, -0.404) | 0.001 | 0 to 1 | 0.091 (-0.384, 0.566) | 0.706 | Shift but never/rarely night shifts | 0.130 (-0.218, 0.478) | 0.464 |
|  | >8 | -0.305 (-0.769, 0.159) | 0.197 | 2 to 3 | 0.114 (-0.084, 0.311) | 0.259 | Some night shifts | -0.206 (-0.687, 0.274) | 0.399 |
|  |  |  |  |  |  |  | Usual/permanentnight shifts | 0.503 (-0.021, 0.985) | 0.410 |
| >50 | <6h | 0.020 (-0.386, 0.427) | 0.922 | 0 to 1 | 0.400 (-0.008, 0.807) | 0.054 | Shift but never/rarely night shifts | -0.181 (-0.482, 0.120) | 0.237 |
|  | >8 | -0.375 (-0.748, -0.001) | 0.049 | 2 to 3 | 0.083 (-0.073, 0.240) | 0.295 | Some night shifts | 0.223 (-0.143, 0.589) | 0.233 |
|  |  |  |  |  |  |  | Usual/permanentnight shifts | -0.412 (-0.903, 0.079) | 0.100 |
| **BMI category** | |  |  |  |  |  |  |  |  |
| ≤25 | <6h | -0.341 (-0.930, 0.247) | 0.255 | 0 to 1 | 0.430 (-0.218, 1.079) | 0.193 | Shift but never/rarely night shifts | -0.211 (-0.588, 0.166) | 0.273 |
|  | >8 | -0.390 (-0.864, 0.084) | 0.107 | 2 to 3 | 0.104 (-0.096, 0.304) | 0.309 | Some night shifts | -0.332 (-0.939, 0.274) | 0.282 |
|  |  |  |  |  |  |  | Usual/permanentnight shifts | 0.002 (-0.641, 0.645) | 0.996 |
| 25-<30 | <6h | -0.477 (-1.008, 0.053) | 0.078 | 0 to 1 | 0.336 (-0.165, 0.838) | 0.189 | Shift but never/rarely night shifts | -0.059 (-0.387, 0.269) | 0.724 |
|  | >8 | -0.369 (-0.834, 0.095) | 0.119 | 2 to 3 | 0.023 (-0.163, 0.209) | 0.810 | Some night shifts | 0.263 (-0.176, 0.702) | 0.240 |
|  |  |  |  |  |  |  | Usual/permanentnight shifts | 0.166 (-0.301, 0.633) | 0.486 |
| ≥30 | <6h | -0.295 (-0.903, 0.314) | 0.341 | 0 to 1 | -0.003 (-0.501, 0.494) | 0.989 | Shift but never/rarely night shifts | 0.567 (-0.020, 1.155) | 0.058 |
|  | >8 | -0.069 (-0.684, 0.547) | 0.827 | 2 to 3 | 0.280 (-0.011, 0.571) | 0.059 | Some night shifts | 0.078 (-0.430, 0.586) | 0.762 |
|  |  |  |  |  |  |  | Usual/permanentnight shifts | 0.129 (-0.642, 0.900) | 0.742 |

CI, Confidence Interval; BMI, Body Mass Index;

| **Supplementary table 13. Multivariate Analysis of Associations Between Sleep Health Dimensions, and Shift Work Status with Prospective Memory Stratified by Sex, Age and BMI** | | | | | | | | | |
| --- | --- | --- | --- | --- | --- | --- | --- | --- | --- |
|  | **Sleep length** | **OR (95%CI)** | ***P* value** | **Sleep quality** | **OR (95%CI)** | ***P* value** | **Sleep Shift** | **OR (95%CI)** | ***P* value** |
| **Sex** |  |  |  |  |  |  |  |  |  |
| Males | <6h | 0.740 (0.441, 1.315) | 0.277 | 0 to 1 | 1.005 (0.59, 1.824) | 0.985 | Shift but never/rarely night shifts | 0.520 (0.356, 0.780) | 0.001 |
|  | >8 | 0.874 (0.490, 1.706) | 0.670 | 2 to 3 | 1.006 (0.794, 1.27) | 0.963 | Some night shifts | 0.944 (0.573, 1.654) | 0.829 |
|  |  |  |  |  |  |  | Usual/permanentnight shifts | 0.836 (0.476, 1.592) | 0.557 |
| Females | <6h | 0.683 (0.410, 1.200) | 0.161 | 0 to 1 | 0.713 (0.406, 1.332) | 0.260 | Shift but never/rarely night shifts | 0.512 (0.362, 0.738) | 0.000 |
|  | >8 | 1.856 (0.982, 3.991) | 0.080 | 2 to 3 | 0.834 (0.663, 1.046) | 0.118 | Some night shifts | 0.581 (0.346, 1.034) | 0.051 |
|  |  |  |  |  |  |  | Usual/permanentnight shifts | 0.526 (0.271, 1.125) | 0.073 |
| **Age** |  |  |  |  |  |  |  |  |  |
| ≤50 | <6h | 0.498 (0.271, 0.998) | 0.035 | 0 to 1 | 0.602 (0.301, 1.349) | 0.181 | Shift but never/rarely night shifts | 0.478 (0.310, 0.766) | 0.001 |
|  | >8 | 1.260 (0.634, 2.881) | 0.544 | 2 to 3 | 0.735 (0.546, 0.984) | 0.040 | Some night shifts | 0.615 (0.359, 1.128) | 0.094 |
|  |  |  |  |  |  |  | Usual/permanentnight shifts | 0.673 (0.364, 1.367) | 0.236 |
| >50 | <6h | 0.827 (0.528, 1.350) | 0.425 | 0 to 1 | 0.939 (0.594, 1.553) | 0.798 | Shift but never/rarely night shifts | 0.532 (0.388, 0.740) | 0.000 |
|  | >8 | 1.293 (0.750, 2.432) | 0.388 | 2 to 3 | 1.005 (0.826, 1.221) | 0.958 | Some night shifts | 0.912 (0.566, 1.551) | 0.719 |
|  |  |  |  |  |  |  | Usual/permanentnight shifts | 0.741 (0.410, 1.459) | 0.349 |
| **BMI category** | |  |  |  |  |  |  |  |  |
| ≤25 | <6h | 0.634 (0.346, 1.249) | 0.161 | 0 to 1 | 0.492 (0.261, 0.999) | 0.037 | Shift but never/rarely night shifts | 0.519 (0.343, 0.807) | 0.002 |
|  | >8 | 0.907 (0.494, 1.837) | 0.769 | 2 to 3 | 0.803 (0.621, 1.038) | 0.095 | Some night shifts | 0.898 (0.447, 2.069) | 0.781 |
|  |  |  |  |  |  |  | Usual/permanentnight shifts | 0.957 (0.431, 2.550) | 0.922 |
| 25-<30 | <6h | 1.007 (0.555, 1.994) | 0.982 | 0 to 1 | 1.326 (0.687, 2.837) | 0.432 | Shift but never/rarely night shifts | 0.450 (0.312, 0.664) | 0.000 |
|  | >8 | 3.951 (1.461, 16.219) | 0.021 | 2 to 3 | 0.97 (0.757, 1.24) | 0.811 | Some night shifts | 0.608 (0.370, 1.051) | 0.060 |
|  |  |  |  |  |  |  | Usual/permanentnight shifts | 0.747 (0.395, 1.569) | 0.402 |
| ≥30 | <6h | 0.475 (0.239, 1.036) | 0.054 | 0 to 1 | 0.889 (0.424, 2.053) | 0.768 | Shift but never/rarely night shifts | 0.801 (0.401, 1.796) | 0.559 |
|  | >8 | 0.791 (0.366, 1.985) | 0.582 | 2 to 3 | 1.080 (0.704, 1.633) | 0.721 | Some night shifts | 0.929 (0.431, 2.325) | 0.862 |
|  |  |  |  |  |  |  | Usual/permanentnight shifts | 0.372 (0.167, 0.949) | 0.024 |

BMI, Body Mass Index; OR, Odds Ratio;

| **Supplementary table 14. Correlation Between Inflammatory Factors and Outcomes of Cognitive Function and Sleep-Related Factors in Multivariable Analysis** | | | | | | | | | | | | |
| --- | --- | --- | --- | --- | --- | --- | --- | --- | --- | --- | --- | --- |
|  | Inflammation markers | | | | | | | | | | | |
|  | Neutct | NeutPct | MonoCt | MonoPct | LymphCt | LymphPct | BasoPct | EosinoPct | LeukCt | CRP | NLR | SII |
| **Cognitive index** |  |  |  |  |  |  |  |  |  |  |  |  |
| Reasoning | 0.0013 | 0.0025 | 0.0003 | 0.0014 | -0.0044^a^ | -0.0033 | -0.0007 | -0.0042 | -0.0011 | -0.0187^c^ | 0.0057^a^ | 0.0078^a^ |
| Reaction time | -0.0138 | -0.0027 | -0.0005 | 0.0158 | -0.0074 | 0.0041 | -0.0513 | -0.0684^a^ | -0.0114 | -0.0673 | -0.0064 | -0.0403^a^ |
| Visual memory | -0.0022 | -0.0002 | -0.0014 | 0.0007 | -0.0008 | 0.0011 | -0.0103 | -0.0009 | -0.0020 | 0.0028 | -0.0013 | -0.0027 |
| Numeric memory | -0.0025 | 0.0027 | -0.0162^a^ | -0.0110 | -0.0111^a^ | -0.0059 | -0.0103 | 0.0143 | -0.0053 | -0.0093 | 0.0086 | 0.0055 |
| Prospective memory | 0.0142 | 0.0185^a^ | -0.0332^a^ | -0.0290^a^ | -0.0133 | -0.0089 | -0.0116 | 0.0173 | -0.0034 | -0.0007 | 0.0274 | 0.0391^a^ |
| **Sleep length** |  |  |  |  |  |  |  |  |  |  |  |  |
| <6 | -0.0075 | -0.0033 | -0.002 | 0.0026 | -0.0086 | -0.0048 | -0.0291 | -0.0074 | -0.0038 | 0.0616 | 0.0011 | 0.0092 |
| >8 | 0.0197 | 0.0038 | 0.016 | 0.0027 | 0.0082 | -0.0079 | 0.0253 | 0.0141 | 0.0158 | 0.0601 | 0.0115 | 0.0019 |
| **Sleep quality** |  |  |  |  |  |  |  |  |  |  |  |  |
| 0 to 1 | -0.0148 | -0.0119 | 0.0203 | 0.0238^a^ | 0.0061 | 0.0091 | 0.0071 | 0.0545^a^ | -0.0016 | 0.1412^c^ | -0.0209 | -0.0125 |
| 2 to 3 | 0.0016 | 0.0015 | -0.0021 | -0.0022 | -0.0010 | -0.0014 | -0.0166 | 0.0087 | 0.0002 | 0.0520^c^ | 0.0026 | 0.0084 |
| **Shift work** |  |  |  |  |  |  |  |  |  |  |  |  |
| Shift but never/rarely night shifts | 0.0016 | 0.0015 | -0.0168 | -0.0022 | -0.0010 | -0.0014 | 0.0054 | -0.0320 | 0.0002 | 0.052^c^ | 0.0026 | 0.0084 |
| Some night shifts | 0.0100 | 0.0057 | -0.0145 | -0.0166 | 0.0051 | 0.0013 | -0.0130 | 0.0007 | 0.0051 | -0.0061 | 0.0048 | 0.0112 |
| Usual/permanent night shifts | 0.0003 | 0.0082 | -0.0180 | -0.0067 | -0.0281^a^ | -0.0202^a^ | 0.0054 | 0.0035 | -0.0078 | 0.0598 | 0.0285 | 0.0241 |
| BasoPct, Basophil Percentage; CRP, C-Reactive Protein;LeukCt, Leukocyte Count; LymphCt, Lymphocyte Count; LymphPct, Lymphocyte Percentage; MonoCt, Monocyte  Count; MonoPct, Monocyte Percentage; NeutCt, Neutrophil Count; NeutPct, Neutrophil Percentage; NLR, Neutrophil-Lymphocyte Ratio; SII, Systemic Inflammation Index; ^a^ *P*<0.05; ^b^ *P* <0.01; ^c^ *P*<0.001 | | | | | | | | | | | | |

| **Supplementary table 15. Two-Sample MR Analysis Results** | | | |
| --- | --- | --- | --- |
| **Method** | **β (95% CI)** | ***P* value** | **SNP** |
| **chronotype and CRP** | |  | 151 |
| MR Egger | -0.15(-0.415, 0.114) | 0.268 |  |
| Weighted median | 0.082(-0.029, 0.193) | 0.147 |  |
| Inverse variance weighted | 0.047(-0.038, 0.132) | 0.275 |  |
| Simple mode | 0.107(-0.221, 0.435) | 0.523 |  |
| Weighted mode | 0.107(-0.242, 0.456) | 0.549 |  |
| **Insomnia and CRP** |  |  | 41 |
| MR Egger | -0.355(-1.025, 0.315) | 0.307 |  |
| Weighted median | -0.306(-0.607, -0.005) | 0.046 |  |
| Inverse variance weighted | -0.023(-0.257, 0.21) | 0.844 |  |
| Simple mode | -0.367(-0.978, 0.243) | 0.248 |  |
| Weighted mode | -0.376(-0.841, 0.09) | 0.124 |  |
| **Snore and CRP** |  |  | 37 |
| MR Egger | -0.879(-2.443, 0.685) | 0.280 |  |
| Weighted median | 0.485(0.091, 0.879) | 0.016 |  |
| Inverse variance weighted | 0.491(0.193, 0.789) | 0.001 |  |
| Simple mode | 0.506(-0.274, 1.285) | 0.213 |  |
| Weighted mode | 0.450(-0.351, 1.252) | 0.280 |  |
| **Sleep length and CRP** |  |  | 66 |
| MR Egger | -0.26(-0.933, 0.413) | 0.453 |  |
| Weighted median | 0.016(-0.226, 0.258) | 0.898 |  |
| Inverse variance weighted | -0.087(-0.264, 0.091) | 0.340 |  |
| Simple mode | 0(-0.517, 0.516) | 0.999 |  |
| Weighted mode | 0.009(-0.379, 0.396) | 0.965 |  |
| **Daytime dozing and CRP** |  |  | 30 |
| MR Egger | -0.607(-1.928, 0.715) | 0.376 |  |
| Weighted median | 0.085(-0.343, 0.516) | 0.698 |  |
| Inverse variance weighted | 0.135(-0.164, 0.433) | 0.376 |  |
| Simple mode | 0.693(-0.229, 1.615) | 0.152 |  |
| Weighted mode | -0.602(-1.53, 0.327) | 0.214 |  |
| **CRP and Reasoning** |  |  | 7 |
| MR Egger | -0.011(-0.021, -0.001) | 0.089 |  |
| Weighted median | -0.006(-0.011, -0.001) | 0.042 |  |
| Inverse variance weighted | -0.005(-0.01,-0.001) | 0.037 |  |
| Simple mode | 0.002(-0.008, 0.011) | 0.729 |  |
| Weighted mode | -0.007(-0.013, -0.001) | 0.083 |  |
| **Snore and Reasoning** |  |  | 32 |
| MR Egger | 0.107(-0.115, 0.33) | 0.355 |  |
| Weighted median | -0.004(-0.038, 0.029) | 0.792 |  |
| Inverse variance weighted | 0.007(-0.021, 0.034) | 0.645 |  |
| Simple mode | -0.02(-0.082, 0.043) | 0.543 |  |
| Weighted mode | -0.015(-0.076, 0.045) | 0.624 |  |

| **Supplementary table 16. Sensitivity Analysis, Including Heterogeneity Test and Horizontal Pleiotropy Test** | | | | | | | |
| --- | --- | --- | --- | --- | --- | --- | --- |
| Heterogeneity test |  |  |  |  |  |  |  |
| Exposure | Outcome | Heterogeneity test (MR-Egger) | | | Heterogeneity test (IVW) | | |
|  |  | Cochrane’s Q | Q (df) | Q (*P* val) | Cochrane’s Q | Q (df) | Q (*P* val) |
| Chronotype | CRP | 178.6883 | 126 | 0.001417923 | 182.0623 | 127 | 0.000988517 |
| Insomnia | CRP | 40.44501 | 29 | 0.07691979 | 41.93918 | 30 | 0.07243607 |
| Snore | CRP | 17.26635 | 29 | 0.957881 | 20.32553 | 30 | 0.9077885 |
| Sleep length | CRP | 82.21689 | 53 | 0.006196583 | 82.64184 | 54 | 0.007313434 |
| Daytime dozing | CRP | 114.9831 | 28 | 1.66322E-12 | 120.1468 | 29 | 4.64703E-13 |
| CRP | Reasoning | 1.639156 | 4 | 0.8017379 | 3.651807 | 5 | 0.6005519 |
| Snore | Reasoning | 32.63214 | 24 | 0.1120762 | 33.71692 | 25 | 0.1140985 |
| Horizontal pleiotropy test |  |  |  |  |  |  |  |
| Exposure | Outcome | Horizontal pleiotropy test (MR-Egger) | | | Horizontal pleiotropy test (MR-PRESSO) |  |  |
|  |  | Intercept | *P* val |  | Global test pval |  |  |
| Chronotype | CRP | 0.003374507 | 0.125471 |  | 0.0006 |  |  |
| Insomnia | CRP | 0.004038511 | 0.309194 |  | 0.0664 |  |  |
| Snore | CRP | 0.01047215 | 0.090861 |  | 0.9186 |  |  |
| Sleep length | CRP | 0.002103622 | 0.602881 |  | 0.0068 |  |  |
| Daytime dozing | CRP | 0.01156251 | 0.271659 |  | 0.0452 |  |  |
| CRP | Reasoning | 0.000524425 | 0.228988 |  | 0.5816 |  |  |
| Snore | Reasoning | -0.000723144 | 0.380615 |  | 0.1118 |  |  |
| Q (df), Q (degree of freedom); IVW, Inverse variance weighting | | | | | | | |

| **Supplementary table 17. Inflammatory Markers Mediating the Association Between Sleep Health Dimensions, Shift Work Status and Cognitive Performance (Imputed Dataset)** | | | | | | | | | | | | |
| --- | --- | --- | --- | --- | --- | --- | --- | --- | --- | --- | --- | --- |
|  |  | PNIE |  |  | TNDE |  |  | TE |  |  | PM |  |
|  |  | Estimate (95%CI) | *P* value |  | Estimate (95%CI) | *P* value |  | Estimate (95%CI) | *P* value |  | Estimate (95%CI) | *P* value |
| **Sleep quality and Reasoning** | | | | | | | | | | | | |
| CRP | 0 to 1 | -0.010 (-0.021,  -0.003) | <0.001 |  | -0.083 (-0.230, 0.074) | 0.282 |  | -0.094 (-0.242, 0.066) | 0.236 |  | 0.060 (-0.618, 1.096) | 0.648 |
|  | 2 to 3 | -0.004 (-0.008,  -0.001) | 0.004 |  | -0.062 (-0.122, -0.006) | 0.038 |  | -0.066 (-0.127, -0.010) | 0.032 |  | 0.036 (-0.006, 0.162) | 0.074 |
| **Sleep quality and Reaction time** | | | | | | | | | | | | |
| EosinoPct | 0 to 1 | 0.000 (-0.000, 0.000) | 0.722 |  | -0.004 (-0.012, 0.004) | 0.398 |  | -0.004 (-0.012, 0.004) | 0.402 |  | 0.247 (-2.024, 2.492) | 0.426 |
|  | 2 to 3 | 0.000 (-0.000, 0.000) | 0.982 |  | -0.003 (-0.006, 0.000) | 0.084 |  | -0.003 (-0.006, 0.000) | 0.086 |  | -0.000 (-0.020, 0.016) | 0.968 |
| **Sleep quality and Prospective memory** | | | | | | | | | | | | |
| MonoPct | 0 to 1 | 1.000 (0.994, 1.006) | 0.970 |  | 0.920 (0.690, 1.276) | 0.622 |  | 0.920 (0.689, 1.277) | 0.62 |  | 0.000 (-0.270, 0.295) | 0.978 |
|  | 2 to 3 | 1.000 (0.997, 1.002) | 0.980 |  | 0.880 (0.787, 0.982) | 0.032 |  | 0.880 (0.788, 0.982) | 0.034 |  | -0.000 (-0.025, 0.018) | 0.996 |
| **Sleep Shift and Reasoning** | | | | | | | | | | | | |
| CRP | Shift but never/rarely night shifts | -0.001 (-0.006, 0.003) | 0.544 |  | -0.226 (-0.333, -0.112) | <0.001 |  | -0.227 (-0.335, -0.113) | <0.001 |  | 0.009 (-0.025, 0.055) | 0.578 |
|  | Some night shifts | -0.003 (-0.009, 0.002) | 0.216 |  | -0.269 (-0.404, -0.137) | <0.001 |  | -0.272 (-0.406, -0.143) | <0.001 |  | 0.008 (-0.023, 0.064) | 0.638 |
|  | Usual/permanent night shifts | -0.000 (-0.006, 0.005) | 0.892 |  | -0.355 (-0.509, -0.190) | <0.001 |  | -0.356 (-0.509, -0.188) | <0.001 |  | -0.000 (-0.024, 0.023) | 0.986 |
| LymphPct | Shift but never/rarely night shifts | -0.000 (-0.003, 0.001) | 0.622 |  | -0.226 (-0.324, -0.116) | <0.001 |  | -0.227 (-0.325, -0.117) | <0.001 |  | 0.005 (-0.015, 0.046) | 0.578 |
|  | Some night shifts | -0.000 (-0.003, 0.002) | 0.680 |  | -0.271 (-0.399, -0.152) | <0.001 |  | -0.271 (-0.399, -0.153) | <0.001 |  | 0.005 (-0.026, 0.047) | 0.768 |
|  | Usual/permanent night shifts | 0.000 (-0.002, 0.003) | 0.954 |  | -0.354 (-0.524, -0.190) | <0.001 |  | -0.354 (-0.525, -0.191) | <0.001 |  | -0.002 (-0.066, 0.063) | 0.988 |
| **Sleep Shift and Numeric memory** | | | | | | | | | | | | |
| LymphPct | Shift but never/rarely night shifts | 0.000 (-0.000, 0.000) | 0.802 |  | -0.055 (-0.082, -0.028) | 0.000 |  | -0.055 (-0.082, -0.028) | 0.000 |  | -0.001 (-0.015, 0.017) | 0.920 |
|  | Some night shifts | -0.000 (-0.000, 0.000) | 0.862 |  | -0.034 (-0.065, -0.002) | 0.040 |  | -0.034 (-0.066, -0.002) | 0.040 |  | -0.004 (-0.087, 0.053) | 0.854 |
|  | Usual/permanent night shifts | 0.000 (-0.000, 0.000) | 0.728 |  | -0.055 (-0.092, -0.017) | 0.004 |  | -0.055 (-0.092, -0.017) | 0.004 |  | -0.013 (-0.087, 0.024) | 0.474 |
| **Sleep quality and Reasoning** | | | | | | | | | | | | |
| CRP | Chronotype | 0.003 (0.001, 0.008) | 0.000 |  | 0.010 (-0.048, 0.067) | 0.920 |  | 0.013 (-0.046, 0.072) | 0.900 |  | 0.240 (-1.336, 0.560) | 0.900 |
|  | Insomnia | 0.000 (-0.001, 0.002) | 0.620 |  | -0.015 (-0.081, 0.027) | 0.580 |  | -0.015 (-0.080, 0.028) | 0.580 |  | -0.079 (-0.473, 0.259) | 0.860 |
|  | Snore | 0.004 (0.001, 0.009) | 0.000 |  | 0.090 (0.029, 0.156) | 0.000 |  | 0.094 (0.036, 0.159) | 0.000 |  | 0.043 (0.016, 0.145) | 0.020 |
|  | Sleep length | -0.000 (-0.001, 0.001) | 0.760 |  | 0.058 (-0.005, 0.102) | 0.080 |  | 0.058 (-0.006, 0.103) | 0.080 |  | -0.013 (-0.101, 0.119) | 0.600 |
|  | Daytime sleepiness | -0.002 (-0.017, 0.012) | 0.820 |  | 0.121 (-0.080, 0.302) | 0.340 |  | 0.119 (-0.065, 0.305) | 0.360 |  | 0.012 (-0.120, 0.236) | 0.560 |
| Abbreviations, CRP, C-Reactive Protein; EosinoPct, Eosinophil Percentage; MonoPct, Monocyte Percentage; LymphPct, Lymphocyte Percentage; PNIE, Pure Natural Indirect Effect; PM, Proportion Mediated; TNDE, Total Natural Direct Effect; TE, Total Effect;   \| **Supplementary table 18. Correlation Between Inflammatory Factors and Outcomes of Cognitive Function and Sleep-Related Factors in Multivariable Analysis Stratified by Sex, Age and BMI** \| \| \| \| \| \| \| \| \| \| \| --- \| --- \| --- \| --- \| --- \| --- \| --- \| --- \| --- \| --- \| \|  \| Sex \| \|  \| Age \| \|  \| BMI \| \| \| \|  \| Male \| Female \|  \| ≤50 \| >50 \|  \| ≤25 \| 25-30 \| ≥30 \| \| **Cognitive index** \|  \|  \|  \|  \|  \|  \|  \|  \|  \| \| Reasoning \| -0.002 \| -0.008 \|  \| -0.003 \| -0.004 \|  \| -0.002 \| -0.014^a^ \| 0.006 \| \| Reaction time \| -0.001 \| 0.001 \|  \| -0.001 \| 0.001 \|  \| 0.001 \| 0.001 \| -0.001 \| \| Visual memory \| 0.010^a^ \| -0.003 \|  \| 0.005 \| 0.002 \|  \| -0.008 \| 0.023^c^ \| -0.007 \| \| Numeric memory \| 0.021^a^ \| -0.017 \|  \| 0.007 \| 0.009 \|  \| 0.002 \| 0.007 \| 0.009 \| \| Prospective memory \| 0.005 \| 0.003 \|  \| -0.000 \| 0.008 \|  \| 0.004 \| -0.002 \| 0.016 \| \| **Sleep length** \|  \|  \|  \|  \|  \|  \|  \|  \|  \| \| <6 \| 0.232 \| 0.018 \|  \| -0.016 \| 0.195 \|  \| 0.307 \| -0.031 \| 0.16 \| \| >8 \| 0.091 \| 0.205 \|  \| 0.067 \| 0.165 \|  \| 0.079 \| 0.347^a^ \| -0.215 \| \| **Sleep quality** \|  \|  \|  \|  \|  \|  \|  \|  \|  \| \| 0 to 1 \| 0.168 \| 0.417^c^ \|  \| 0.428 ^c^ \| 0.251^b^ \|  \| 0.257 \| 0.207^a^ \| 0.526^b^ \| \| 2 to 3 \| 0.038 \| 0.085 \|  \| 0.053 \| 0.073 \|  \| 0.076 (0.131) \| 0.116 ^a^ \| -0.055 \| \| **Shift work** \|  \|  \|  \|  \|  \|  \|  \|  \|  \| \| Shift but never/rarely night shifts \| -0.089 \| -0.074 \|  \| -0.138 \| -0.034 \|  \| -0.062 \| -0.128 \| 0.025 \| \| Some night shifts \| 0.236 \| 0.059 \|  \| -0.039 \| 0.292 ^a^ \|  \| 0.238 \| 0.05 \| 0.196 \| \| Usual/permanent night shifts \| 0.039 \| -0.008 \|  \| -0.100 \| 0.13 \|  \| 0.005 \| 0.017 \| 0.034 \| \| a, *P* < 0.05; b, *P* < 0.01; c, *P* <0.001 \|  \|  \|  \|  \|  \|  \|  \|  \|  \|  \| **Supplementary table 19. Inflammatory Markers Mediating the Association Between Sleep Health Dimensions, Shift Work Status and Cognitive Performance Stratified by Sex, Age and BMI** \| \| \| \| \| \| \| \| \| \| \| \| \| \| --- \| --- \| --- \| --- \| --- \| --- \| --- \| --- \| --- \| --- \| --- \| --- \| --- \| \|  \|  \| PNIE \|  \|  \| TNDE \|  \|  \| TE \|  \|  \| PM \|  \| \|  \|  \| Estimate (95%CI) \| *P* value \|  \| Estimate (95%CI) \| *P* value \|  \| Estimate (95%CI) \| *P* value \|  \| Estimate (95%CI) \| *P* value \| \| **Sleep quality and Reasoning** \| \| \| \| \| \| \| \| \| \| \| \| \| \| CRP \| 0 to 1 \| -0.004 (-0.010, -0.002) \| < 0.001 \|  \| -0.021 (-0.180, 0.232) \| 0.600 \|  \| -0.025 (-0.183, 0.226) \| 0.600 \|  \| 0.170 (-0.093, 0.146) \| 0.6 \| \|  \| 2 to 3 \| -0.001 (-0.003, 0.001) \| 0.800 \|  \| -0.078 (-0.190, -0.066) \| < 0.001 \|  \| -0.078 (-0.190, -0.066) \| < 0.001 \|  \| 0.011 (-0.013, 0.034) \| 0.8 \| \| **Sleep length and Reasoning** \| \| \| \| \| \| \| \| \| \| \| \| \| \| CRP \| < 6 \| -0.001 (-0.005, 0.005) \| 0.800 \|  \| -0.325 (-0.529, -0.148) \| < 0.001 \|  \| -0.326 (-0.533, -0.145) \| < 0.001 \|  \| 0.002 (-0.023, 0.013) \| 0.800 \| \|  \| > 8 \| -0.004 (-0.010, -0.000) \| < 0.001 \|  \| -0.106 (-0.356, 0.020) \| 0.400 \|  \| -0.109 (-0.365, 0.013) \| 0.400 \|  \| 0.034 (-0.516, 0.195) \| 0.400 \| | | | | | | | | | | | | |


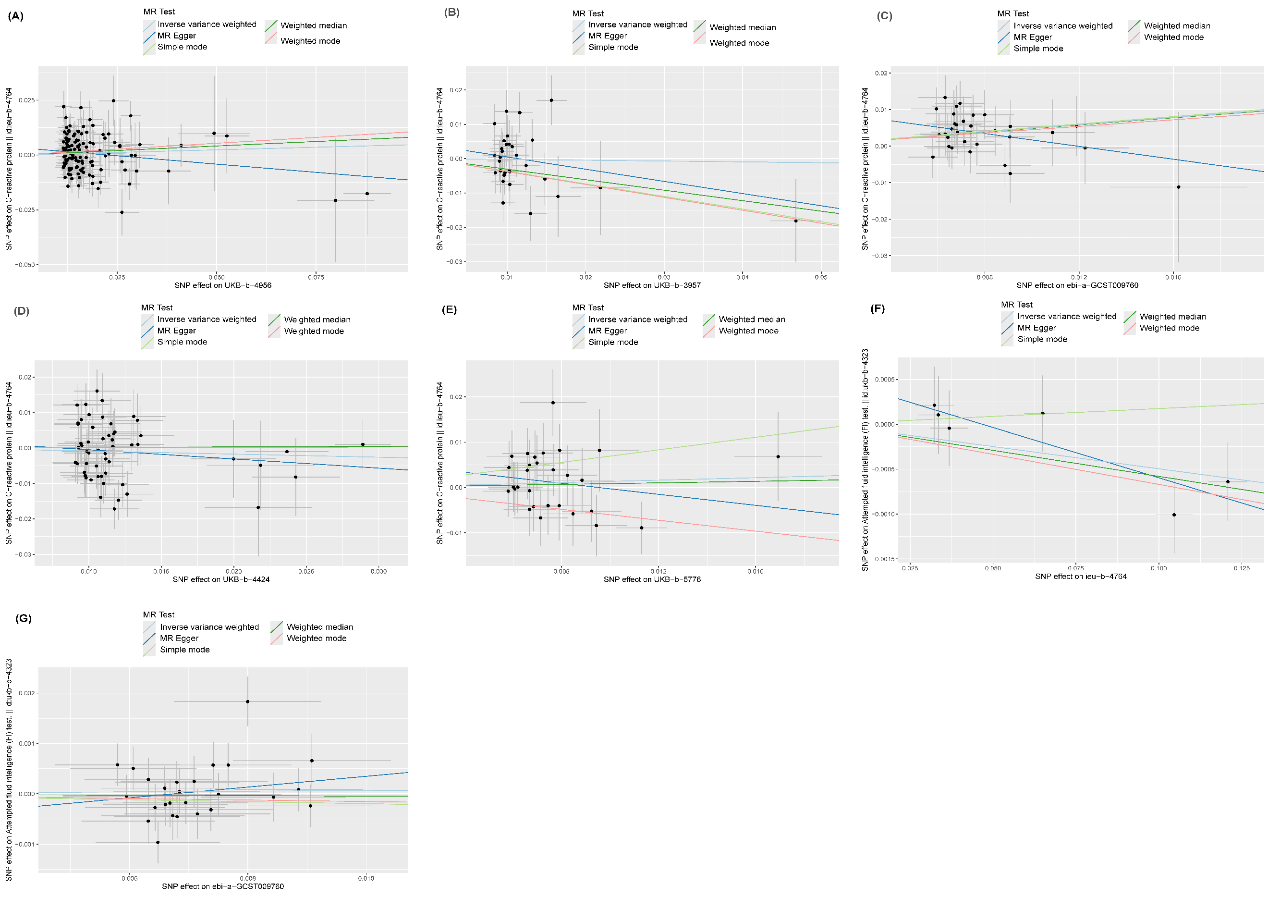


**Supplementary figure 1. Scatter Plots of Genetic Correlation Between Sleep Quality, CRP and Reasoning by Different MR Analysis Methods.**

A, Chronotype and CRP; B, Insomnia and CRP; C, Snore and CRP; D, Sleep length and CRP; E, Daytime dozing and CRP; F, CRP and Reasoning; G, Snore and Reasoning.


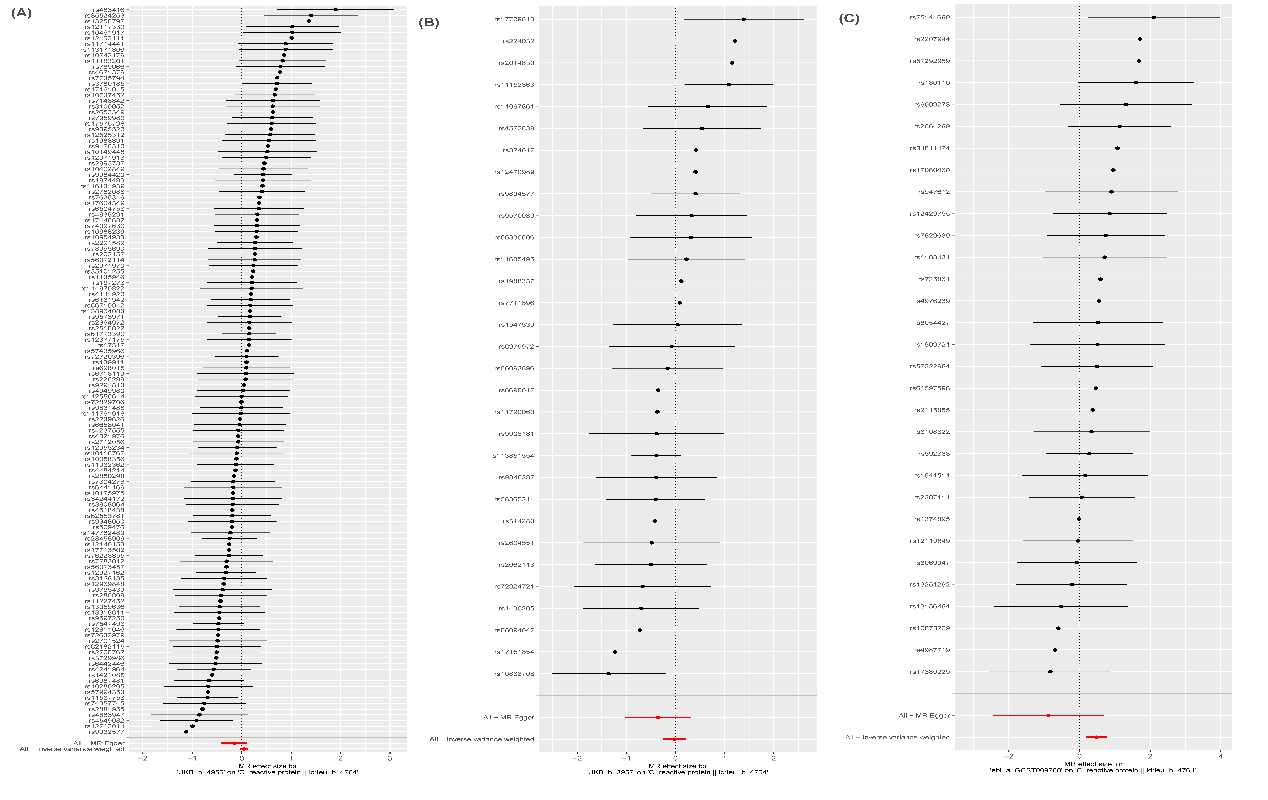


**Supplementary figure 2. “Leave-one-out” results of genetic correlation between sleep quality, CRP and reasoning by different MR analysis methods.**

A, chronotype and CRP; B, Insomnia and CRP; C, Snore and CRP; D, Sleep length and CRP; E, Daytime dozing and CRP; F, CRP and Reasoning; G, Snore and Reasoning.


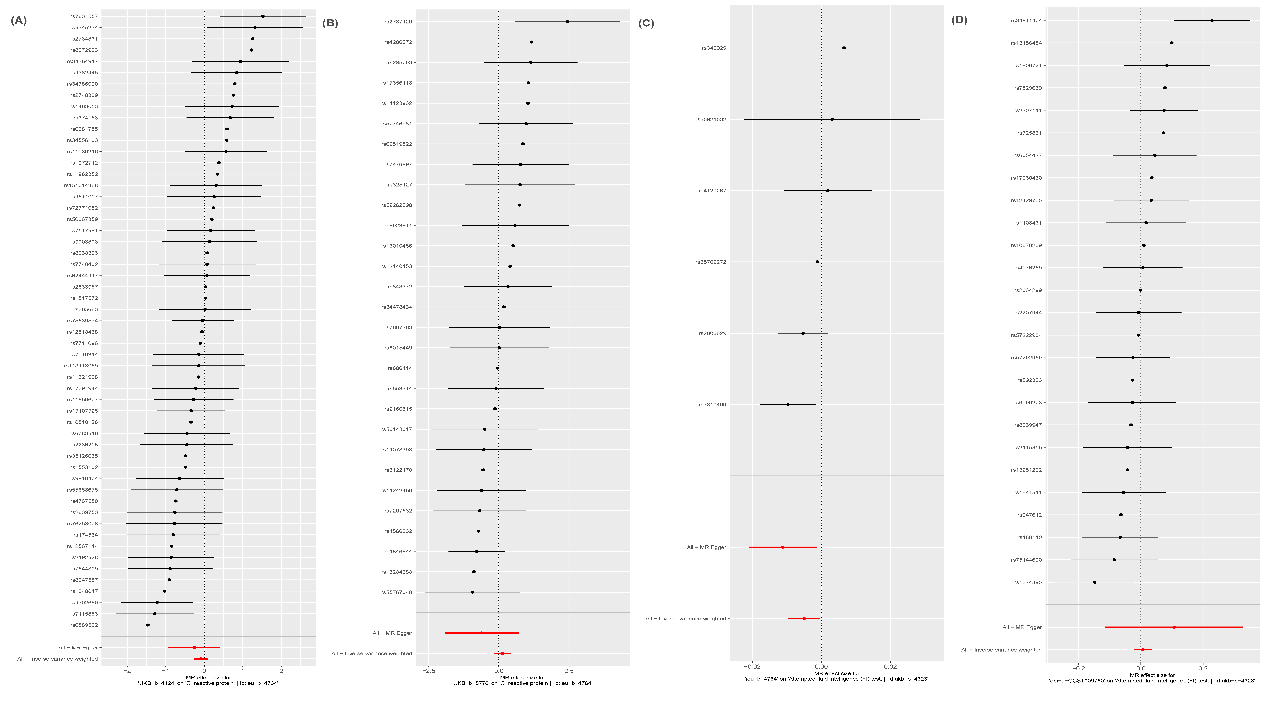


**Supplementary figure 3. “Leave-One-Out” Results of Genetic Correlation Between Sleep Quality, CRP and Reasoning by Different MR Analysis Methods.**

A, Sleep length and CRP; B, Daytime dozing and CRP; C, CRP and Reasoning; D, Snore and Reasoning.

**Supplementary Note 1. Details of UK Biobank Assessment**

**Sleep** was evaluated from three aspects: sleep length, sleep quality and shift work^1,2^.

**Cognitive function**^3^ was evaluated from five aspects: reasoning, reaction time, visual memory, numeric memory and prospective memory.

**Reasoning**: Participants were given two minutes to complete 13 verbal and numerical reasoning problems. The primary outcome measure was the total number of correct answers, ranging from 0 to 13.

**Reaction time**: Participants were asked to complete a timed symbol-matching test analogous to the card game Snap. They were instructed to press a response button upon detecting matching symbol pairs. The outcome variable was the mean reaction time across 12 trials containing matched pairs. Due to skewed data distribution, values were log-transformed (using natural logarithm ln[x]) and multiplied by -1. Thus, shorter reaction times yielded higher scores. These data were presented as “Adjusted Reaction Time” in tables and figures to indicate transformation-derived metrics.

**Visual memory**: Six pairs of symbol cards were randomly arranged face up before participants, then turned face down. Participants were asked to memorize card pair locations and match them with minimal errors. The primary outcome was the number of errors per participant (range: 0 to 146). Due to skewed distribution with excess zeros, data were log-transformation (natural logarithm of [x + 1], ln[x+1]). Visual memory scores were multiplied by -1 so that fewer errors yielded higher values. These data were presented as “Adjusted Visual Memory Score” in tables and figures to reflect the transformation protocol.

**Numeric memory**: Participants were first shown a two-digit number on-screen and instructed to memorize it. After the number disappeared following a brief pause, they entered it via keyboard. The next number increased by one digit upon correct recall of the previous one. The outcome was the maximum digit span length (range: 0 to 12 digits) correctly recalled. This task was administered at baseline and T2, but not at T1.

**Prospective memory**: In this task, participants were instructed to remember to execute a preplanned instruction after a delay filled with assessment activities. At the beginning of the test session, they received the following directive: ‘At the end of the games, we will show you four colored symbols and ask you to touch the blue square. However, to test your memory, we actually want you to touch the orange circle.’ Responses were coded as correct (score = 1) if participants touched the orange circle on their first attempt, and incorrect (score = 0) if they touched the blue square.

**Supplementary Note 2. Details of Inflammation-Related Biomarkers**

Neutrophil-to-lymphocyte ratio^4-6^ (NLR) and systemic immune-inflammation index (SII) were analyzed. NLR, calculated as the absolute neutrophil count divided by absolute lymphocyte count, served as an inflammation biomarker. Prior studies have established associations between NLR and cognitive function in certain disease contexts. The systemic immune-inflammation index^6^ (SII), a composite biomarker of systemic inflammation, was calculated as: platelet count × neutrophil count / lymphocyte count. In all analyses, levels of inflammatory biomarkers were transformed using the natural logarithm.

**Supplementary Note 3. Details of Covariates**

Smoking and alcohol status were classified based on whether responses were “never”, “current” or “previous” for these covariates. Socioeconomic status was categorized by relying on this variable, specifically the Townsend deprivation index (UK Biobank), with reference to the national census output for each postal code area.

**Supplementary Note 4. Indicators of mediation analysis**

**The Pure Natural Indirect Effect (PNIE)** was quantified using the CMAverse package in R. This indicator represented the component of the total effect of the exposure on the outcome that was transmitted exclusively through the mediator variable. This effect estimate isolated the portion of the indirect effect attributable solely to the pathway from the exposure to the outcome via the mediator, under the hypothetical condition where the exposure influenced the outcome only through the mediator, while holding the direct path constant.

**Total Natural Direct Effect (TNDE)** was quantified the aggregate effect of the exposure on the outcome that operated directly, without transmission through the mediator variable. This effect encompassed all potential direct pathways linking the exposure to the outcome. The TNDE represented the net effect of the exposure on the outcome after controlling for the influence of the mediator, reflecting the direct association between the exposure and the outcome. Importantly, the TNDE captured the direct association independent of any intermediate role played by the mediator variable.

**Total Effect (TE)** was represented the overall effect of the exposure on the outcome, encompassing the sum of the direct effect and the indirect effect. It quantified the total magnitude of change in the outcome associated with a change in the exposure. The TE integrated both the indirect influence of the exposure transmitted through the mediator variable and its direct influence on the outcome. Thus, the TE provided a comprehensive measure of the strength and direction of the total relationship between the exposure and the outcome.

**Proportion Mediated (PM)** was defined as the proportion of the total effect attributable to the indirect effect, specifically quantifying the relative importance of the mediator in the relationship between the exposure and the outcome. It was calculated as the ratio of the Pure Natural Indirect Effect (PNIE) to the Total Effect (TE). PM values ranged between 0 and 1. Higher PM values indicated a stronger explanatory role of the mediator, signifying that a larger proportion of the exposure's overall effect on the outcome operates through the mediator pathway.

**Supplementary Note 5. Details of mediation analysis in mendelian randomization**

The mediation effect (Beta) was calculated as the product of the regression coefficient for the association between the independent variable and the mediator variable (Beta (X→Z)) multiplied by the regression coefficient for the association between the mediator variable and the dependent variable (Beta (Z→Y)). The proportion of the mediation effect relative to the total effect was expressed as: R = [Mediation effect (Beta) / Regression coefficient for the association between the independent variable and the dependent variable (Beta (X→Y))] × 100%. After adjustment for confounding factors, the effect of the exposure on the outcome was considered the direct effect. This direct effect was calculated as the regression coefficient for the association between the independent variable and the dependent variable (Beta (X→Y)) minus the mediation effect (Beta). Bootstrap methods were used to estimate confidence intervals and assess the statistical significance of the mediation effect.

**Supplementary Note 6. Details of the Mendelian randomization**

In the MR analysis, each instrumental variable (IV) was selected based on stringent criteria to align with the foundational hypothesis of causal inference: (1) single nucleotide polymorphisms (SNPs) should be strongly associated with the exposure(s) of interest; (2) SNPs should be independent of any confounding factors; (3) SNPs should affect the outcome only through the exposure(s) of interest. 38 SNPs meeting a genome‐wide significance threshold (P < 5 × 10‐8) were considered potential IVs. Subsequently, disequilibrium (LD) variants, and palindromic SNPs were removed.

Furthermore, SNPs were selected based on several criteria (1) minimal likelihood of linkage disequilibrium (r ^2^ > 0.001, distance = 10,000 kb); (2) sufficient IV strength, as evaluated the by F statistic > 10 using the following formula:

$$F=\left( \frac{N-K-1}{2K} \right)*\left( \frac{R^{2}}{{1-R}^{2}} \right)$$

(3) lack of strong association with the outcome (P > 0.01); (4) not associated with other confounding phenotypes (searched GWAS catalog, with threshold of *P* < 1× 10^‐5^). SNPs not present in the outcome data were substituted with available proxy SNPs (r ^2^ > 0.8), identified through LDlink. Instrumental variables (IVs) comprised 151, 41, 37, 66, and 30 SNPs for sleep phenotypes, insomnia, snoring, sleep duration, and daytime drowsiness, respectively. Seven SNPs served as IVs for CRP and 32 for snoring in causal assessments with reasoning performance.

**Supplementary references:**

1. Fan M, Sun D, Zhou T, et al. Sleep patterns, genetic susceptibility, and incident cardiovascular disease: a prospective study of 385 292 UK biobank participants. *European heart journal*. Mar 14 2020;41(11):1182-1189. doi:10.1093/eurheartj/ehz849

2. Kanki M, Nath AP, Xiang R, et al. Poor sleep and shift work associate with increased blood pressure and inflammation in UK Biobank participants. *Nature communications*. Nov 4 2023;14(1):7096. doi:10.1038/s41467-023-42758-6

3. Ell J, Schiel JE, Feige B, et al. Sleep health dimensions and shift work as longitudinal predictors of cognitive performance in the UK Biobank cohort. *Sleep*. Jun 13 2023;46(6)doi:10.1093/sleep/zsad093

4. Liang J, Guan X, Sun Q, Hao Y, Xiu M. Neutrophil/lymphocyte ratio and cognitive performances in first-episode patients with schizophrenia and healthy controls. *Progress in neuro-psychopharmacology & biological psychiatry*. Dec 20 2024;135:111092. doi:10.1016/j.pnpbp.2024.111092

5. Contaldi E, Magistrelli L, Cosentino M, Marino F, Comi C. Lymphocyte Count and Neutrophil-to-Lymphocyte Ratio Are Associated with Mild Cognitive Impairment in Parkinson's Disease: A Single-Center Longitudinal Study. *Journal of clinical medicine*. Sep 22 2022;11(19)doi:10.3390/jcm11195543

6. Wang M, Zeng X, Liu Q, Yang Z, Li J. The association between sleep duration and cognitive function in the U.S. elderly from NHANES 2011-2014: A mediation analysis for inflammatory biomarkers. *Journal of affective disorders*. Apr 15 2025;375:465-471. doi:10.1016/j.jad.2025.01.154
